# Ordinary and Extraordinary Movement Behaviour of Small Resident Fish within a Mediterranean Marine Protected Area

**DOI:** 10.1371/journal.pone.0159813

**Published:** 2016-07-20

**Authors:** Eneko Aspillaga, Frederic Bartumeus, Cristina Linares, Richard M. Starr, Àngel López-Sanz, David Díaz, Mikel Zabala, Bernat Hereu

**Affiliations:** 1 Departament d’Ecologia, Universitat de Barcelona, Barcelona, Spain; 2 Centre d’Estudis Avançats de Blanes (CEAB-CSIC), Girona, Spain; 3 Centre de Recerca Ecològica i Aplicacions Forestals (CREAF), Cerdanyola del Vallès, Spain; 4 ICREA, Barcelona, Spain; 5 Moss Landing Marine Laboratories, University of California Sea Grant Extension Program, Moss Landing, California, United States of America; 6 Institut de Ciències del Mar (ICM-CSIC), Barcelona, Spain; 7 Centre Oceanogràfic de les Balears, Instituto Español de Oceanografía, Palma de Mallorca, Spain; Department of Agriculture and Water Resources, AUSTRALIA

## Abstract

It is important to account for the movement behaviour of fishes when designing effective marine protected areas (MPAs). Fish movements occur across different spatial and temporal scales and understanding the variety of movements is essential to make correct management decisions. This study describes in detail the movement patterns of an economically and commercially important species, *Diplodus sargus*, within a well-enforced Mediterranean MPA. We monitored horizontal and vertical movements of 41 adult individuals using passive acoustic telemetry for up to one year. We applied novel analysis and visualization techniques to get a comprehensive view of a wide range of movements. *D*. *sargus* individuals were highly territorial, moving within small home ranges (< 1 km^2^), inside which they displayed repetitive diel activity patterns. Extraordinary movements beyond the ordinary home range were observed under two specific conditions. First, during stormy events *D*. *sargus* presented a sheltering behaviour, moving to more protected places to avoid the disturbance. Second, during the spawning season they made excursions to deep areas (> 50 m), where they aggregated to spawn. This study advances our understanding about the functioning of an established MPA and provides important insights into the biology and management of a small sedentary species, suggesting the relevance of rare but important fish behaviours.

## Introduction

Understanding the movement ecology of fishes is crucial for the management of marine ecosystems. The efficacy of a marine protected area (MPA) to protect and restore overexploited fish populations within its boundaries and to enhance sustainable fishing activities in adjacent areas depends greatly upon the relationship between the size of the MPA and the scale of the movements of targeted fish species [1,2]. However, movements of juvenile and adult fishes can occur over several spatial and temporal scales, providing a challenge when designing effective MPAs [3]. Therefore, having good baseline information on space-use patterns of different fish species is essential for making effective spatial management decisions, such as the design of an MPA or the configuration of a reserve network [4,5].

Home range (HR) is the area in which an individual spends the majority of its time and undertakes most of its routine activities, such as foraging and resting [1,2]. Very sedentary or territorial species have small HRs, and are more likely to benefit from the establishment of spatial protection [6,7] than highly mobile or migratory species that, by expending more time in open areas, become vulnerable to being fished [8,9].

In addition to regular or routine movements, many sedentary species undergo sporadic movements involving larger spatial scales, responding to special needs raised by physical and biological factors. For example, many species undertake migrations to specific breeding areas during the spawning season [10,11]. In coral reefs, these migrations often result in fish spawning aggregations (FSAs) [12], which are highly predictable both in time and in space, and therefore render those species particularly susceptible to being overfished [13,14]. While about 200 tropical fish species are known to form FSAs [12], very little is known about spawning behaviours of fish species in temperate seas. For instance, in the Mediterranean Sea there is only one species, the dusky groper (*Epinephelus marginatus*), which has been confirmed to form FSAs [15,16].

An understanding of the variety of fish movement patterns, from HR-level ordinary movements to sporadic (or extraordinary) migrations, is needed to correctly assess the effectiveness of MPAs. Acoustic telemetry techniques have proven to be powerful tools to serve this purpose, allowing long-term monitoring of fish movements over a wide range of spatial scales [17]. During the last two decades, these techniques have been successfully applied around the world to study the movements of a broad variety of marine species [17,18].

Here, we focus on the white seabream *Diplodus sargus* (L., 1758), using acoustic telemetry to study the movements of a common necto-benthic species within different protection levels of a Mediterranean MPA. *D*. *sargus* is one of the most abundant species of the infralittoral zone in the Mediterranean Sea, with a high ecological relevance as a grazer and prey species that helps shape rocky marine ecosystems [19–21]. It is also of great importance in artisanal and recreational fisheries [22,23]. *D*. *sargus* is a well-studied species, and many aspects of its biology have been widely described [24–26]. Several telemetry studies have been published that describe the movements of *D*. *sargus* in different environments, such as coastal lagoons [27] and artificial reefs [28–31]. More recently, three studies focused on the movements of this species in relation to MPAs [32–34]. All these studies describe the high sedentariness of *D*. *sargus*, reporting small HRs and high site fidelity. Some of the studies also describe daily movement cycles for this species [28–31,34], but little is known about how daily movement behaviour is affected during extreme environmental conditions or in the spawning period, the latter known to occur from March to June.

In this study we performed one of the longest telemetry experiments conducted to date with *D*. *sargus* in natural environments. Our high resolution and fine scale spatial data provide a comprehensive and up-to-date view of the movement ecology of this species, one that describes diel movement patterns and movement behaviour during severe climatic events and spawning periods. Information gathered from the wide spectra of movement scales and ecological conditions studied provide novel insights for the conservation of the species, and more generally, for the management of benthic fishes in Mediterranean MPAs.

## Materials and Methods

### Study area

**Fig 1:**
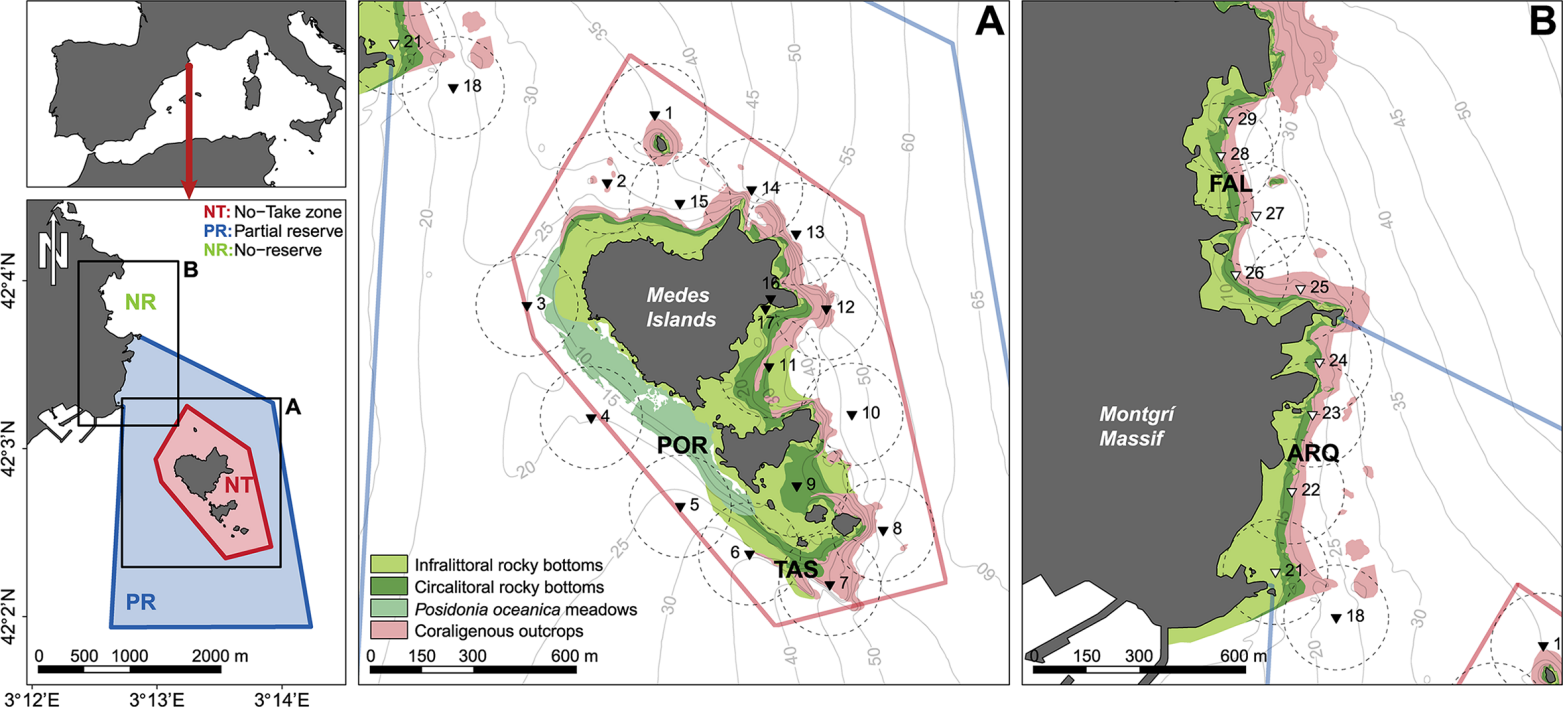
Study area and the acoustic receiver array. Black triangles correspond to acoustic receivers placed in May 2007 and white triangles to the receivers placed in September 2008. Dotted circles represent the average detection range (150 m). Red bold letters stand for the *Diplodus sargus* capture locations: TAS, Tascons; POR, Portitxol; ARQ: Arquets; FAL: Falaguer. The topographic base map (1:5.000) and the sea bottom bionomic map [35] are freely accessible through the Institut Cartogràfic i Geològic de Catalunya (www.icgc.cat) under Creative Commons Attribution License (CC BY 4.0).

The study was carried out in the Medes Islands MPA (Catalonia, NW Mediterranean Sea), which comprises three zones with different protection levels (Fig 1). The fully protected marine reserve or no-take zone (NT) is placed in the small archipelago of the Medes Islands, and was established in 1983. A partially protected buffer zone or partial reserve (PR) encompasses a section of the nearby coast of the Montgrí massif. In this zone, limited traditional artisanal fishing (longline and trammel net gear) and recreational angling are allowed with restrictions. The rest of the coast is not subject to any specific regulation; all type of activities, including spearfishing, are allowed, and hence is considered a no-reserve zone (NR).

The Medes Islands and the Montgrí coast are areas of high ecological value because of the high diversity of marine habitats they encompass [35]. The shallow zones closest to the land contain heterogeneous rocky habitats, which are followed by coralligenous outcrops in deeper zones. In waters deeper than about 50 m, hard bottoms are succeeded by soft sandy bottoms, which form a band of soft sediments about 800 m wide between the rocky habitats of the Medes islands and the Montgrí massif on the coast. Since the establishment of the Medes Islands MPA, several studies have reported higher abundance and biomass of *D*. *sargus* and other vulnerable species, such as the dusky grouper *Epinephelus marginatus* and the common dentex *Dentex dentex*, inside the NT zone than in the PR and NR zones [36,37].

In this area of the coast, winter storms that arrive from the north and east are frequent [38]. One exceptionally severe easterly storm arrived off the Catalan coast on 26–27 December 2008. The storm produced winds surpassing 85 km/h and waves up to 14.4 m in maximum height. This storm, known as the St. Steve’s Day Storm, greatly affected the study area, causing profound impacts on benthic communities at depths of up to 20 m [39].

### Acoustic monitoring system

A network comprised of 27 moored acoustic receivers (VR2 and VR2W, VEMCO, Nova Scotia, Canada) was installed in the study area (Fig 1). Moorings included anchors, chain, line, and subsurface floats. Receivers were placed 8 m below the surface. The installation of the receivers was performed in two stages. A first set of 17 receivers was placed within the NT zone, covering the entire perimeter of the Medes Islands, in June 2007 (Fig 1A). A second set of 10 receivers was installed in the Montgrí coast in September 2008, 5 of them in the PR zone and 5 in the NR zone (Fig 1B). All moorings and receivers were removed in July 2009, with the exception of #8, which was lost in the beginning of the experiment due to adverse sea conditions and was not replaced. During the extreme storm of 2008, receiver #22 was lost and then replaced by the receiver in position #23 in January 2009.

Signal range-tests were performed in the study site by placing multiple receivers at different distances from a transmitter of the same model used to tag fish. These tests revealed that the probability of detecting a signal was >90% out to a range of 150 m, after which the probability of reception dropped below 50% (S1 Fig). No test-transmitters were used during the study to detect possible changes in the reception efficiency. It has been described that the detection probabilities can be highly variable in coastal waters due to environmental noise caused by wave action, physical impediments and biological activity [40]. We considered this impediment when interpreting our data, and hence we have avoided drawing biological conclusions from temporal patterns in the number of receptions.

### Fish tagging

#### Ethics statement

The tagging protocol was approved by the Committee on the Ethics of Animal Experimentation of the University of Barcelona. The Department of Environment of the Catalan Government granted permissions for fishing, operating and releasing the animals in the Medes Islands Marine Reserve. All surgery was performed under 2-phenoxyethanol anaesthesia, and all efforts were made to minimize suffering.

Individuals of *D*. *sargus* where caught and tagged with V13P-1H acoustic tags (dimensions: 48 x 13 mm, power output: 153 dB, weight in water: 6.5 g; VEMCO, Nova Scotia, Canada), programmed to produce signals at random delay times between 80 and 180 s. A traditional angling technique was used to catch the individuals from the shoreline, and barbless hooks were used to minimize injuries. Fish were anesthetized by dipping them in a 0.2 ml·l^-1^ 2-phenoxyethanol solution. Tags were surgically introduced in the peritoneal cavity through an incision of 2 cm in the ventral area, which was then closed with sterile surgical staples. Before being released, fish were placed in recovery tanks filled with clean seawater until a full recovery of their normal activity was observed (usually between 10–20 min). The tagging procedure was conducted under aseptic conditions, and all efforts were made to minimize animal stress and suffering. The tagging methodology used is a standard procedure that has been used on *D*. *sargus* and other species in previous studies [28,41,42]. The procedure has been demonstrated to have no long-term, adverse effects on fish behaviour and survival [43].

A total of 41 individuals of *D*. *sargus* between 25 and 35 cm length were successfully tagged and released in four different locations (Fig 1). Two groups of 11 and 9 individuals, respectively, were caught in May 2007 in two locations separated by about 300 m within the NT zone (TAS and POR). In September and October 2008, two additional groups of 14 and 7 individuals were tagged in the ARQ (PR zone) and FAL (NR zone) locations. In order to assess the homing ability of this species, seven of the individuals captured in the ARQ location within the PR zone in the Montgrí coast were released in the TAS location within the Medes islands NT zone. The rest of the animals were released in the same location as they were captured.

### Data analysis

Data from the VR2 receivers were regularly downloaded to VUE software (VEMCO, Nova Scotia, Canada) and directly imported to R (v.3.2.1) [44], where all the pre-processing and data analyses were performed. Sole receptions in a single receiver within a 24 h time interval were considered spurious and were therefore deleted.

#### Residence index

A residence index (RI) was calculated for each fish by dividing the total number of days the fish was detected (DD) by the number of days in the entire tracking period (TP) [45]. The time interval during which receiver #22 was missing (see above) was not used to calculate the RI of the fish from the ARQ location.

#### Home range size

The Brownian Bridge Movement Model (BBMM) [46] was used to compute the utilization distribution (UD), i.e. the probability distribution defining the animal’s use of space [47], of each fish for the whole tracking period. The BBMM has advantages over the classical kernel UD estimator. While the kernel UD estimator only takes into account the spatial distribution of the locations, the BBMM also considers their time dependence, assuming a conditional random walk movement model between pairs of locations. Furthermore, the BBMM incorporates the location error in an implicit way during the calculation. Those two advantages make the BBMM especially suitable to analyse data from passive acoustic telemetry, where the position of the reception is fixed and the location error is as large as the signal detection range. BBMMs have been already applied to acoustic telemetry experiments [42,48]. BBMM were applied using the BBMM package (v.3.0) for R [49].

HR and core area sizes were calculated as the minimum areas encompassing the 95% and 50% of the UD estimate volumes, respectively. We measured the space use sharing between each pair of *D*. *sargus* individuals using the Utilization Distribution Overlapping Index (UDOI) suggested by Fieberg & Kochanny [50]. The UDOI, being a function of the UD and its uniformity, equals zero when two UDs do not overlap, equals 1 when the UDs are completely overlapped and are uniformly distributed, and >1 when two UDs which are not uniformly distributed show a high degree of overlap [50]. HR and core area sizes and the UDOI were calculated with the adehabitatHR package (v.0.4.14) for R [51].

#### Diel patterns

In order to study the circadian behaviour of *D*. *sargus*, receptions were classified into day and night time periods as defined by local sunset and sunrise time from the US Naval Observatory (http://aa.usno.navy.mil/data/index.php, data accessed in 2013/09/12 for the coordinates of the Medes Islands 42°03'N 3°13'E). The presence of diel patterns was assessed for each individual following two different approaches. First, we created spatial chronogram plots, which were visually inspected. Spatial chronogram plots represent the receivers with the largest number of receptions in 30 min intervals in each day of the tracking period, and are an effective way to visualize, on a fine temporal scale, presences and absences of an individual among different zones of the receiver array. In a second approach, diel changes in the position of the fish were inferred from the mean depth and hourly reception number data (i.e. total number of receptions received during a single phase divided by its duration). We defined a phase-transition value (PT) for each day to night transition (D-N) as follows:

$${PT}_{D-N}=D_{t}-N_{t}$$

where *D*_*t*_ is the corresponding value of a given variable for a day phase and *N*_*t*_ is the value of the same variable for the consecutive night phase. The distribution of the *PT*_*D-N*_ values gives us a comprehensive view of the repeatability and cyclical nature of the diel movements. For example, significantly negative *PT*_*D-N*_ values calculated with the mean depth indicate repeated movements to deeper waters at the nightfall, significantly positive values indicate movements to shallower waters, and values that do not differ from 0 indicate that there is no a consistent pattern in the change of the fish depth. *PT*_*D-N*_ values for the hourly reception number provides information about diel movements between zones that differ in their acoustic performance. In order to ensure the cyclical nature of these movements, we also analysed the distribution of the PT values computed for pairs of consecutive day phases (D-D):

$${PT}_{D-D}=D_{t}-D_{t+1}$$

where *D*_*t+1*_ is the value of the variable in the next consecutive day-phase.

#### Extraordinary movements

Sporadic extraordinary movements where inferred from the daily percentages of fish that were detected at given depths and distances from the edge of the HR (95% boundary). We generated depth classes using 1 m depth-intervals and distance-to-HR classes in categories of 200 m. Then, we computed on a daily basis the percentage of fish that were observed in each depth and distance-to-HR class. These percentages were then used to separate the daily observations into two categories: ordinary days, that is, days with ordinary movement behaviour, and extraordinary days, that is, days with extraordinary movement behaviour representing clear statistical outliers of the spatial and temporal metrics used. The two categories were grounded on a hierarchical clustering analysis and visualized with a metric multidimensional scaling, based on Bray-Curtis dissimilarities, and performed with the vegan package (v.2.3–2) for R [52]. Finally, the occurrence of extraordinary movements was visually compared to *in situ* seawater temperature data (hourly measures for the Medes Islands, provided by the T-MedNet network, http://www.t-mednet.org/) and local wave height data (daily measures gently provided by J. Pascual, http://meteolestartit.cat).

## Results

### Summary of receptions

**Fig 2:**
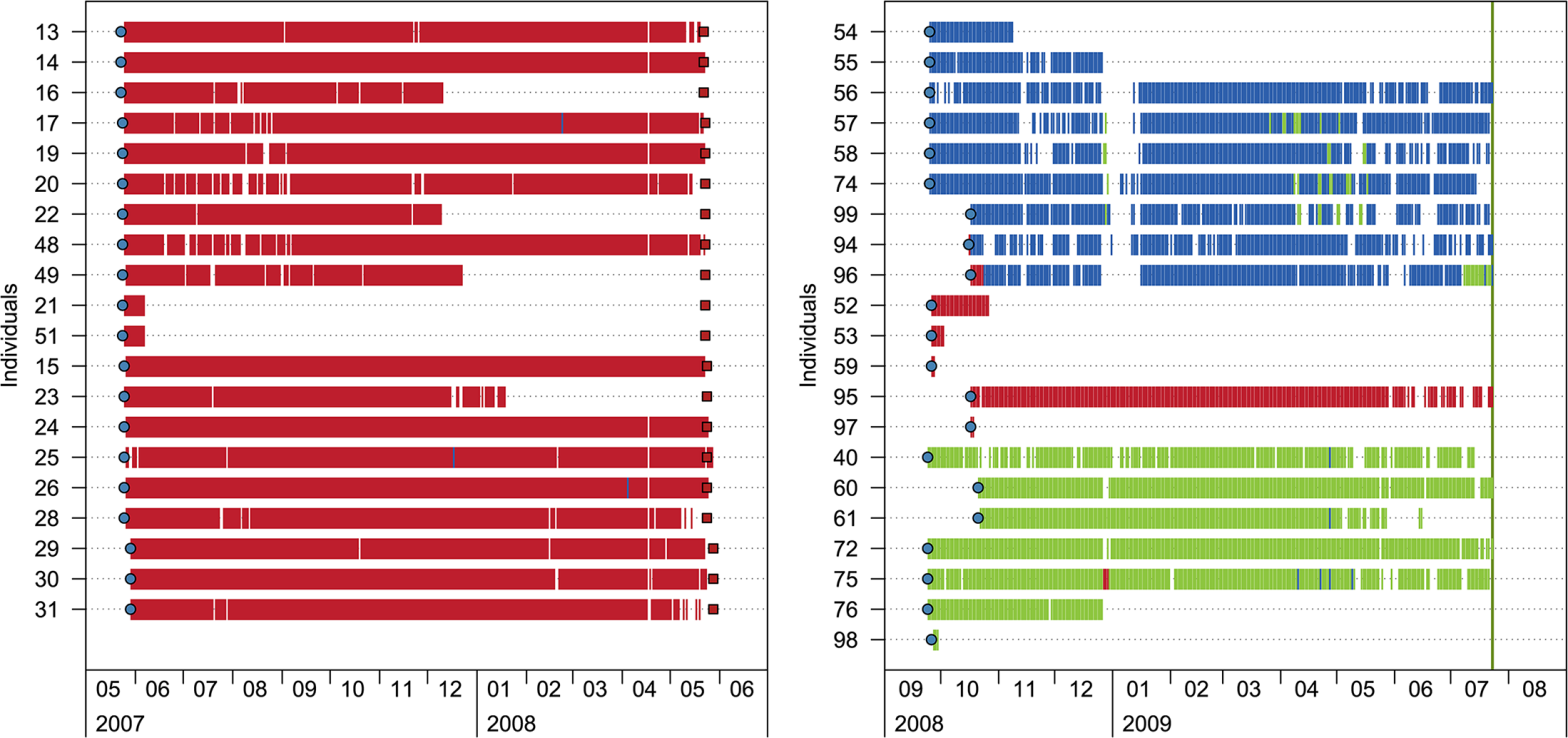
Daily presence-absence plot of tagged *Diplodus sargus* within the monitored area. Colours represent the different protection zones of the MPA; Red: no-take zone (NT), Blue: partial reserve (PR), Green: no-reserve (NR). Fish tag and release dates (blue circles), expected transmitter battery dead dates (red squares) and the final withdrawal of the receiver array (vertical green line) are shown.

A total amount of 816,520 valid receptions were recorded by the receiver array during the whole monitoring period (Table 1). Long tracking periods were registered for almost all individuals, 329 ± 65 d (mean ± SD) for fish caught in the NT zone (TAS and POR locations) and 219 ± 88 d for those caught in the PR and NT zones (ARQ and FAL locations) (Fig 2). Three fish (#21, #51 and #98) disappeared within the first 15 monitoring days and were not detected again throughout the experiment, so they were not considered in the following analysis. Two fish (#55 and #76) did not return to their HR after the extreme storm of 2008.

**Table 1:** Summary of the monitoring information of tagged *Diplodus sargus*. DD: Detection Days; TP: total Tracking Period (d); RI: Residence Index, CA: Core Area; HR: Home Range.

Fish ID	Length (cm)	Capture location	Capture date	Recep. No.	DD	TP	Depth Range (m)	RI NT	RI PR	RI NR	CA 50% (km²)	HR 95% (km²)
13	22	TAS	2007-05-23	21767	353	361	0.7–8.6	0.98	0	0	0.08	0.41
14	32	TAS	2007-05-23	93053	363	364	3.2–12.9	1	0	0	0.16	0.67
16	21	TAS	2007-05-23	9605	193	200	0.2–10.4	0.96	0	0	0.08	0.40
17	29	TAS	2007-05-24	28459	353	363	4.0–13–0	0.97	0	0	0.14	0.61
19	26	TAS	2007-05-24	48800	357	364	0–15.0	0.98	0	0	0.16	0.67
20	33	TAS	2007-05-24	13348	329	356	1.6–17.3	0.92	0	0	0.18	0.76
22	26	TAS	2007-05-24	16582	197	199	0–9.1	0.99	0	0	0.19	0.86
48	23	TAS	2007-05-24	30983	344	364	0.2–10.1	0.95	0	0	0.18	0.74
49	23	TAS	2007-05-24	14388	201	212	0–4.0	0.95	0	0	0.18	0.83
21*	21	TAS	2007-05-24	850	13	13	-	-	-	-	-	-
51*	28	TAS	2007-05-24	1835	13	13	-	-	-	-	-	-
15	29	POR	2007-05-25	56314	363	364	0.4–9.5	1	0	0	0.14	0.81
23	30	POR	2007-05-25	10423	229	239	0–14.2	0.96	0	0	0.08	0.46
24	24	POR	2007-05-25	50621	364	366	0.1–9.3	0.99	0	0	0.10	0.57
25	26	POR	2007-05-25	46071	361	369	0.4–6.6	0.98	0	0	0.24	1.01
26	29	POR	2007-05-25	61909	364	366	0.2–13.6	0.99	0	0	0.11	0.68
28	33	POR	2007-05-25	19962	342	356	0.4–6.4	0.96	0	0	0.11	0.51
29	32	POR	2007-05-29	23692	356	360	0–7.3	0.99	0	0	0.18	0.74
30	26	POR	2007-05-29	32047	356	361	0.3–9.4	0.99	0	0	0.12	0.73
31	29	POR	2007-05-29	22679	343	357	0.7–9.9	0.96	0	0	0.25	1.01
54	29	ARQ	2008-09-25	3772	45	45	0–14.6	0	1	0	0.05	0.22
55	26	ARQ	2008-09-25	2020	47	48	0–5.5	0	0.98	0	0.06	0.27
56	35	ARQ	2008-09-25	9859	211	237	0–5.1	0	0.89	0	0.05	0.24
57	29	ARQ	2008-09-25	12768	230	235	0.4–9.8	0	0.97	0.04	0.05	0.26
58	35	ARQ	2008-09-25	8595	202	235	0.4–15.0	0	0.86	0.02	0.06	0.30
74	29	ARQ	2008-09-25	14961	220	228	0–7.3	0	0.96	0.04	0.06	0.28
99	29	ARQ	2008-10-17	9003	165	213	0–8.2	0	0.76	0.04	0.06	0.32
94T	26	ARQ	2008-10-16	7972	165	216	0.4–11.7	0	0.76	0	0.09	0.35
96T	23	ARQ	2008-10-17	5521	196	215	0.4–9.3	0.03	0.85	0.07	0.06	0.32
52T†	32	ARQ	2008-09-26	10197	31	31	-	-	-	-	-	-
53T*	28	ARQ	2008-09-26	297	7	7	-	-	-	-	-	-
59T*	25	ARQ	2008-09-26	74	2	2	-	-	-	-	-	-
95T†	23	ARQ	2008-10-17	48957	256	280	-	-	-	-	-	-
97T*	26	ARQ	2008-10-17	42	2	2	-	-	-	-	-	-
40	23	FAL	2008-09-24	14054	244	293	0.6–11.4	0	0.00	0.83	0.05	0.20
60	32	FAL	2008-10-21	16194	267	276	0–9.6	0	0.00	0.97	0.04	0.18
61	21	FAL	2008-10-21	9938	211	219	0–8.9	0	0.00	0.96	0.04	0.17
72	21	FAL	2008-09-24	20098	295	303	0.6–10.6	0	0.00	0.97	0.05	0.21
75	24	FAL	2008-09-24	14720	278	301	0.7–14.9	0.01	0.01	0.92	0.06	0.24
76	23	FAL	2008-09-24	4013	93	94	1.3–12.8	0	0.00	0.99	0.04	0.16
98*	35	FAL	2008-09-26	77	3	4	-	-	-	-	-	-

*Letter ‘T’ accounts for translocated fish that were released in TAS*. Fish marked with a ‘*’ disappeared from the study site, *and those marked with a ‘*†*’ were considered dead*.

None of the seven translocated fish remained in the released location. Two individuals (#94 and #96) returned to their original location after time intervals of 12 h and 6 d, respectively, and remained there until the end of the experiment (Fig 2). Three individuals (#53, #59 and #97) were detected for 7 d in the NT zone, but then departed from the area covered by the receptors and were never detected again in either the PR nor NR zones (Fig 2). The two remaining translocated fish (#52 and #95) were considered dead, as they were detected in the NT zone over long periods (31 and 257 d, respectively) by a single receiver and at a constant depth.

### Site fidelity and home range sizes

All fish were full-time residents, presenting high RI values within their zones (Mean ± SD = 0.95 ± 0.06) (Table 1), with no significant differences among fish from different locations (Kruskal-Wallis test, p > 0.05). No relationship was detected between RI and fish size or tracking period length (Kruskal-Wallis test, p > 0.05). Fish were detected within narrow bathymetric ranges; the 95% of all receptions were registered between 0.4 and 11 m depth (Table 1).

**Fig 3:**
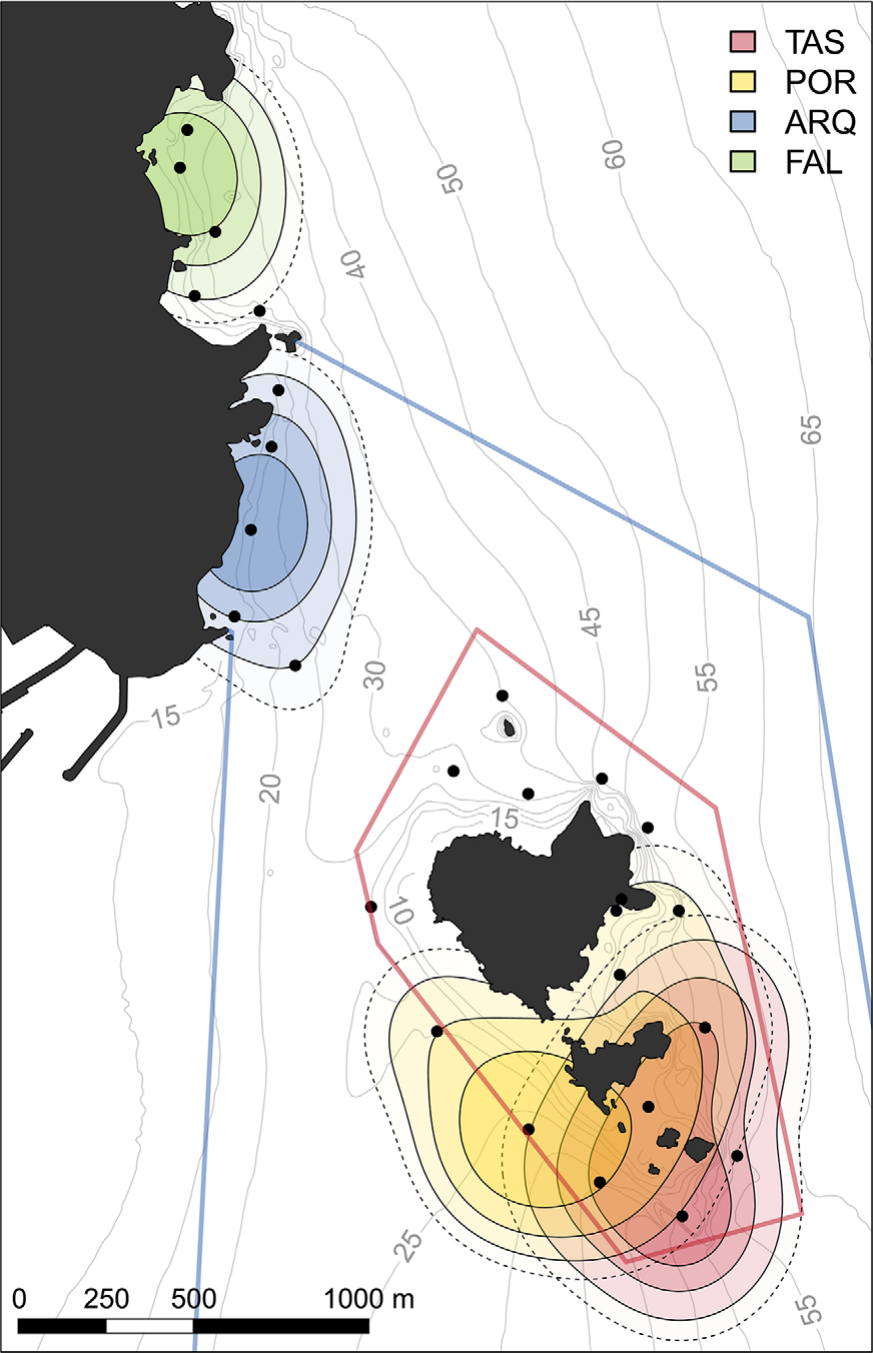
Mean spatial Utilization Distribution estimates of *Diplodus sargus*, for each capture location. The concentric polygons represent, from inside out, the areas covering the 50% (core area), 75%, 90%, and 95% (home range) of the volume of the Utilization Distribution computed by the Brownian Bridge Movement Model. The topographic base map (1:5.000) is freely accessible through the Institut Cartogràfic I Geològic de Catalunya (www.icgc.cat) under Creative Commons Attribution License (CC BY 4.0).

**Fig 4:**
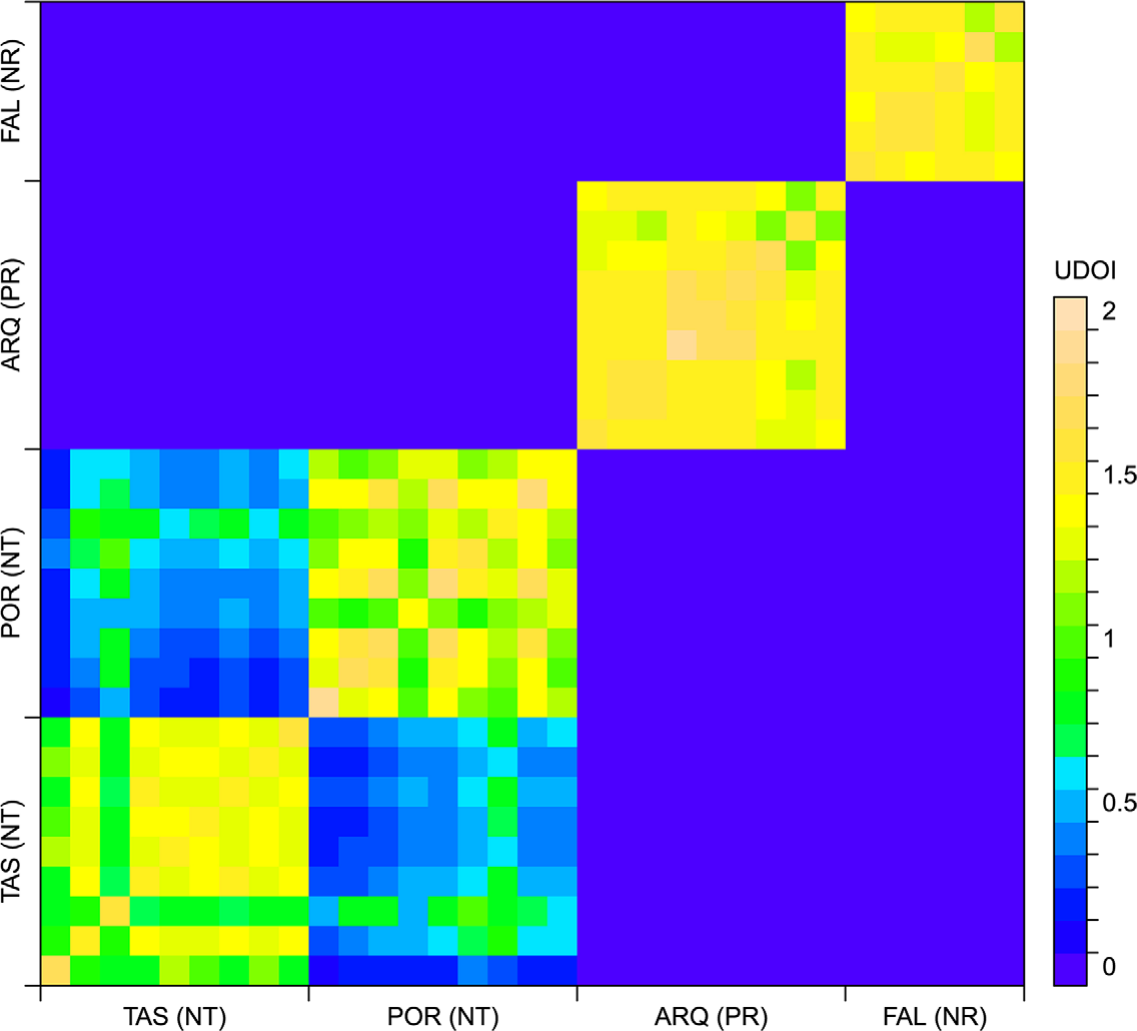
Utilization Distribution Overlap Index (UDOI) calculated for each pair of *Diplodus sargus* individuals. Yellow blocks represent high UDOIs observed when comparing individuals captured in the same location. Green and blue blocks represent lower degrees of overlap and the purple areas represent no-overlapping HRs.

All fish resided in core areas close to their capture location (Fig 3). HR sizes ranged between 0.16 and 1.01 km^2^ and core area sizes ranged between 0.04 and 0.25 km^2^ (Table 1). HR sizes were significantly smaller in fish from PR and NR zones compared to fish from the NT zone (one-way ANOVA, p < 0.01) (Fig 3), presumably due to a difference in the spatial arrangement of the receivers. No effect of fish length or tracking period on the HR size was observed (one-way ANOVA, p > 0.05). The UDOI values denoted a very high degree of HR overlap between individuals from the same location (UDOI > 1) (Fig 4). The UDOI was lower (< 0.7) when comparing individuals from the two locations within the NT zone (TAS and POR). There was no HR overlap (UDOI = 0) when comparing individuals that came from different zones of the reserve.

### Diel patterns

**Fig 5:**
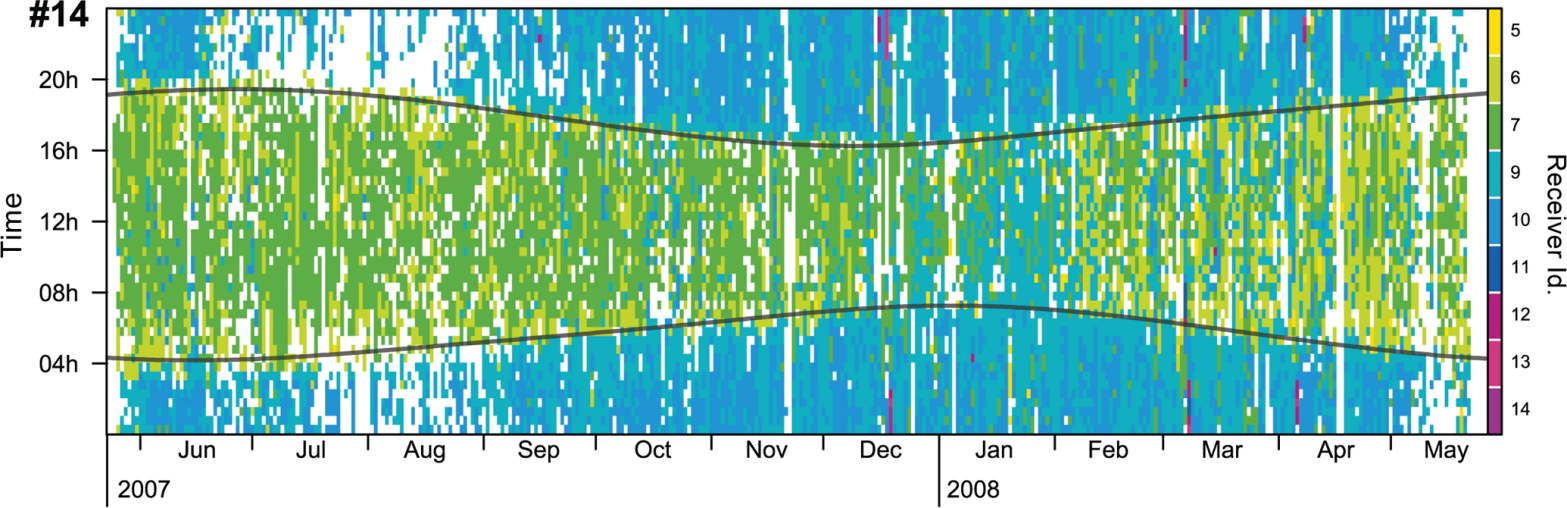
Spatial chronogram plot for one tagged *Diplodus sargus* individual (#14), showing a clear diel movement pattern. This individual was observed at receivers 5, 6 and 7 during the day (green areas) and moved to receivers 9 and 10 during the night. Some punctual excursions to receivers 12, 13 and 14 are visible in December, March and April. White areas represent 30 min intervals with no receptions. See Fig 1 for the placement of the receivers.

Visual inspection of spatial chronogram plots revealed clear diel patterns in 70% of tagged individuals (n = 23). These patterns were characterized by different colours either representing changes in the detecting receivers or reception gaps (white areas). An example of a spatial chronogram plot showing a clear daily pattern can be seen in Fig 5. Spatial chronogram plots for the rest of individuals are available as Supporting Information (S2 Fig).

**Fig 6:**
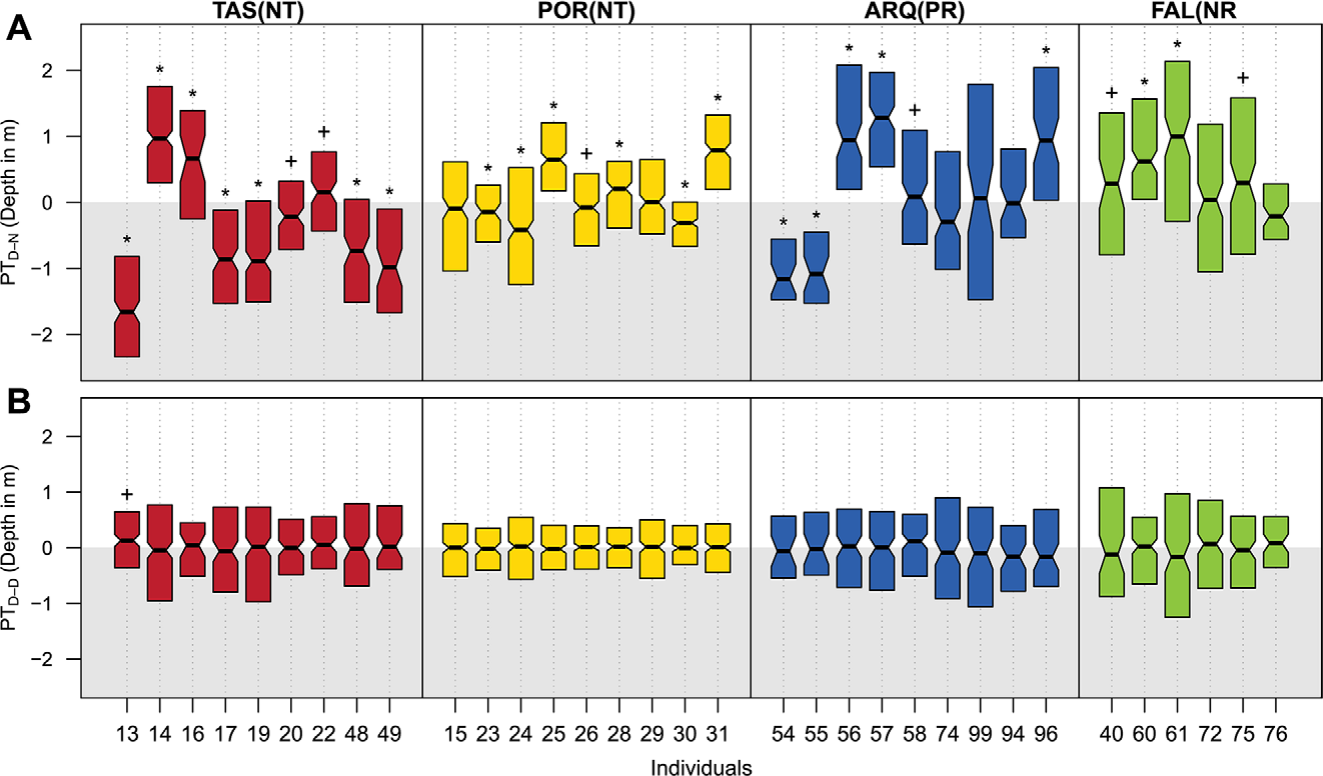
**Distribution of phase-transition values (PT) calculated for the mean depth between consecutive day-night (A) and day-day (B) phases, for each *Diplodus sargus* individual.** Lower and upper boundaries of the boxes represent the first and third quantiles of value distributions. Significant results of the Wilcoxon signed-rank tests against the null hypothesis of a median of zero are expressed by the symbols above the boxes; +: p < 0.05; *: p < 0.01.

The phase-transition values calculated for the mean depth between day and night phases (*PT*_*D-N*_) revealed diel movements in depth in 70% of tagged individuals (n = 23), for which the median of the values was significantly different from zero (Wilcoxon signed-rank test, p < 0.01) (Fig 6A). In contrast, the medians of the phase-transition values between consecutive day phases (*PT*_*D-D*_) did not significantly differ from zero in any fish (Wilcoxon signed-rank test, p > 0.05)–with the exception of the slight deviation observed in individual #13 (Wilcoxon signed-rank test, p < 0.05)–(Fig 6B), which means that each fish tends to be at a similar depth during day phases and confirms the cyclical nature of these movements. However, this analysis revealed large individual-level heterogeneity on depth daily-pattern behaviours: 30% of individuals (n = 10) were found at deeper areas during the night than during the day, while 39% of individuals (n = 13) showed the inverse pattern. Phase transition values for the hourly reception number showed the same heterogeneity (S3 Fig). In this case, 48% of fish (n = 16) had significantly more receptions during the night and 42% individuals (n = 14) had more receptions during the day. Bringing all the results together, 100% of the monitored *D*. *sargus* individuals showed the signature of diel patterns based on at least one of the different metrics used: zone, depth and reception number.

### Extraordinary movements

**Fig 7:**
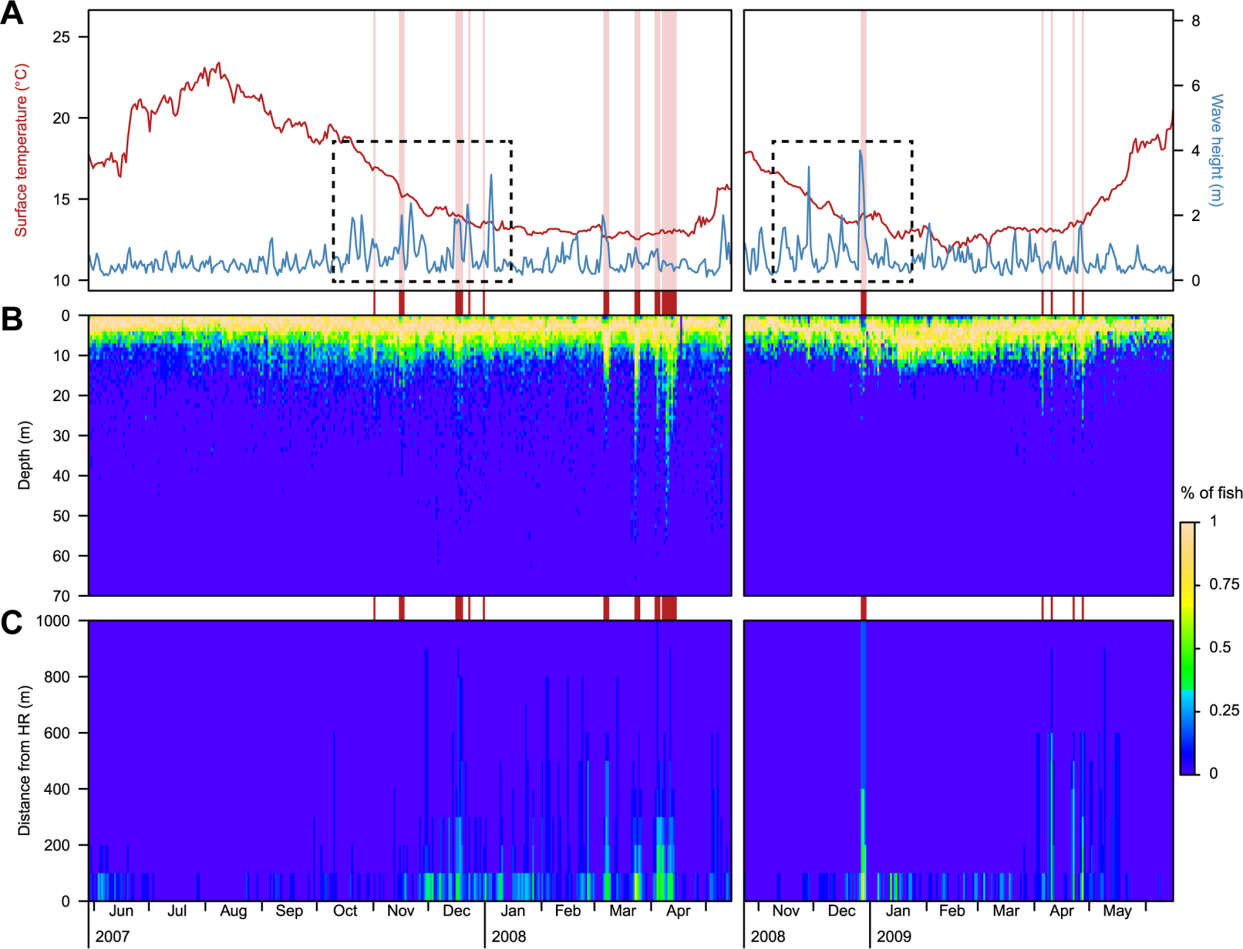
**Daily mean sea surface temperature and wave height (A) and percentages of fish observed at different depths (B) and distances from their HR boundary (C).** Red lines in the back point out days in which extraordinary activities were detected by the cluster analysis. Dotted black rectangles highlight periods with recurrent high wave conditions.

**Fig 8:**
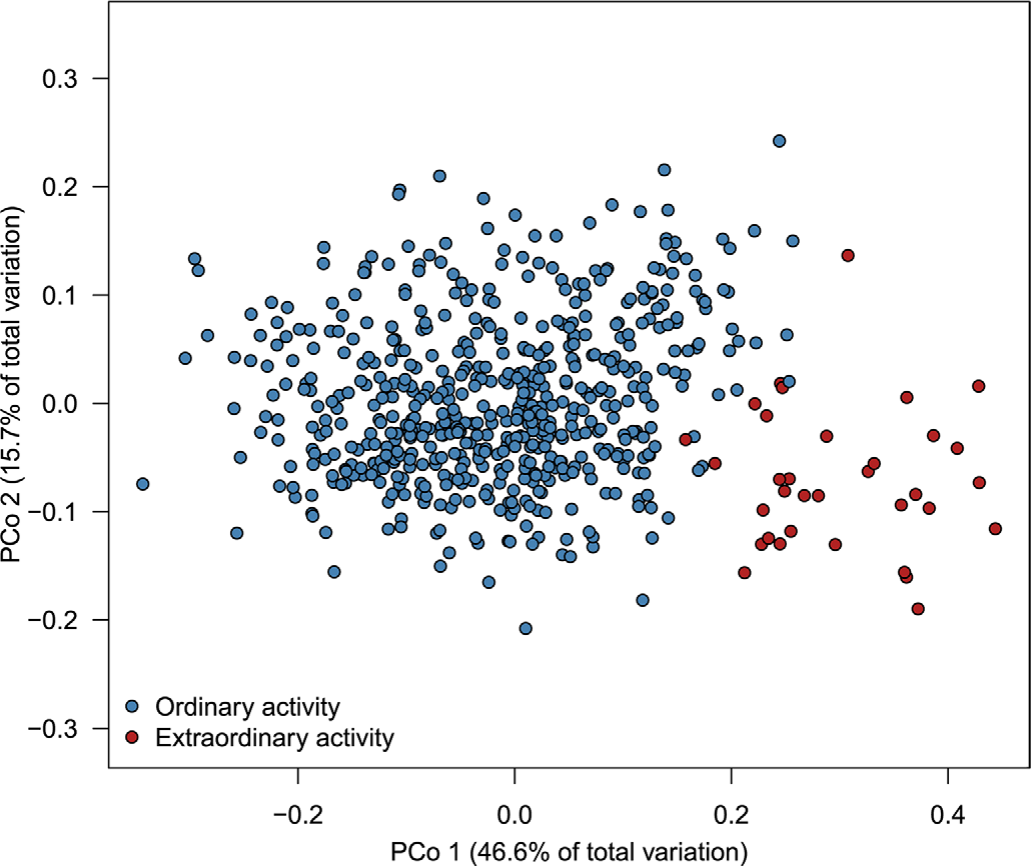
Metric multidimensional scaling analysis depicting similarities between days in fish occurrence at different depths and distances-to-HR. The two main groups of days detected by the cluster analysis (ordinary and extraordinary) are shown in different colours.

Based on the daily occurrence of *D*. *sargus* at different depths and distances from their individual HR (95% boundary), the cluster analysis (see Methods section) was able to distinguish between two consistent day typologies: one for days with ordinary activity (n = 551, 94% of the analysed days) and another for days representing extraordinary behaviours (n = 34, 6% of the analysed days) (Figs 7 and 8). Extraordinary days were characterized mainly by the presence of an elevated percentage of individuals (> 50%) at deeper than normal depths (usually below 20 m), and to a lesser degree, by the presence of individuals (> 30%) at greater distances from the HR boundary. All the days with extraordinary behaviours occurred between November and December and March and April in the two study periods: 2007–2008 (corresponding to fish from the NT zone) and 2008–2009 (fish from the PR and NR zones).

Days with extraordinary movements happening from November to December coincided with (or were close to) periods with large (and highly variable) swell conditions (Fig 7). In contrast, extraordinary movements detected from March to April, could not be related to storm events but coincided with the spawning season for *D*. *sargus* [53,54].

Storms caused a few individuals to move large distances from their HRs (but they were not necessarily observed at greater depths). The most extreme movement was performed by a single individual (#75) during the extreme storm of 2008, which travelled more than 2 km back and forth from its HR boundary (set in the NR zone), and was detected by several receivers from the NT zone (Fig 7; see the spatial chronogram plot for the individual #75 in S2 Fig).

Extraordinary movements observed during the spawning period involved large numbers of individuals moving to greater depths. During these days, 100% (n = 13) and 46% (n = 6) of the individuals tracked in the NT zone where observed deeper than 20 m and 50 m, respectively. For the same period, in the PR and NR zones vertical movements up to 20 m (not deeper most likely due to the position of the receivers, see Fig 1) were detected in 75% of individuals (n = 9). Likewise, fish were observed swimming large horizontal distances from their HR during the spawning period. In the NT zone, 50% of individuals (n = 7) were seen more than 400 m further from the edge of their HR, mainly traveling to the northern part of the Medes Islands (receivers #1, #2, #13 and #14) (e.g. see spatial chronogram plots for fish #14, #20 and #26 in S2 Fig). In the PR and NR zones, 42% of individuals (n = 5) surpassed the distance of 400 m from their HR, crossing the reserve boundary between the PR and NR zones (e.g. see spatial chronogram plots for fish #57, #74, and #75 in S2 Fig).

## Discussion

### Estimating sedentariness and HR sizes

Our study confirms the highly residential nature of *D*. *sargus*, and provides new evidence about the extent of this sedentary behaviour. Our estimate of mean HR (0.49 ± 0.26 km^2^) falls within those reported by previous studies (between 0.03 and 4 km^2^) [28–31,33]. Nevertheless, our findings suggest that this HR could be even smaller. For example, we found that the capture location of each individual was highly consistent with the placement of the HR; fish caught in the same location shared almost their entire estimated HR (UDOI values > 1), while the overlap among fish from different locations was very low, even if the locations were separated for only 300 m. Moreover, the bathymetric distribution of the tagged fish was restricted to relatively shallow depths, rarely exceeding 15 m. This distribution coincides with descriptions of the diurnal behaviour of this species provided by Sala & Ballesteros [26], who reported the highest densities of this species near the surge zone, where it feeds on algae and benthic invertebrates.

The narrow bathymetric range and the low degree of HR overlap between individuals that were captured in the two close locations within the NT zone, illustrate the high territoriality of this species, but also point out that the estimated size of the HR is, in all likeliness, overestimated. When contrasting the extension of the estimated HR of each individual, the bathymetry of the study site [35], and the depth distribution of tagged individuals it became evident that a major part of the estimated HR falls in deep areas that are rarely or never frequented by the animals. In fact, in our study only 9–27% of the area of the HR is located in depths above 15 m. Thus, the real HR of this species must be dramatically smaller than the estimated one.

The overestimation of the HR is due to the large positional uncertainty of the passive acoustic telemetry incorporated in the estimation of the UD. This uncertainty depends on the detection radius of the acoustic transmitters and the characteristics of the study site and may range between tens and hundreds meters. When including this positional error as the kernel bandwidth in kernel density functions [55] or as the location error in the BBMM [46], the resulting utilization probabilities might spill to areas that are in fact not frequented by the animals. This HR overestimation is inherent to every acoustic telemetry study that applies error-scaled UD estimation methods, but it may only be apparent in steep study areas, presenting large depth variations within short distances, such as our study site. The development of new modelling techniques that take into account the depth information from the transmitters and the bathymetrical data of the study site would allow the acquisition of more accurate and non-overestimated utilization probabilities of the animals. Moreover, models incorporating benthic topography will better identify habitat selection behaviour and diel fine-scale movements of fishes, with clear implications for the management of the species at small spatial scales within the MPA.

Our data as well as several behavioural descriptions of *D*. *sargus* [26,56,57] suggest that the small HR of this species is divided in two smaller areas or microhabitats: a feeding area during the day and a resting or sheltering area during the night, with two small migrations between them. The repetitive day-night patterns observed throughout the study period and the variability found between individuals suggest that day and night activity spots remained constant over the long-term and are specific for each individual.

Inferring nocturnal and diurnal activity patterns based on the number of receptions is highly problematic [40]. Local abiotic and biotic factors can drastically reduce the reception performance of acoustic signals [58–60]. Therefore, the specific placement of the day-time foraging and night-time resting territories in acoustically more or less favourable areas will generate different reception patterns, which might not be related to periods with different degrees of biological activity. Therefore, the strong territoriality of *D*. *sargus* has major implications for the interpretation of temporal patterns from acoustic telemetry data. For this reason, and due to the lack of test signals, we have avoided drawing behavioural conclusions from patterns in the number of receptions, and they are only presented as an additional confirmation of the patterns observed in both the spatial chronogram plots and the mean depth changes between day and night phases.

Our results demonstrate that even relatively small MPAs might be extensive enough to effectively protect *D*. *sargus* populations. Moreover, due to the high site fidelity of this species, parameters such as density, mean size and biomass of *D*. *sargus* populations could be used as meaningful indicators of local pressures and ecological status at each study site [61]. Therefore, we can expect that the management activities performed in each zone of the MPA, such as reducing the fishing pressure, will have a significant impact on those indicators. Several studies performed in the Medes Islands marine reserve have reported significantly higher biomasses, densities and mean sizes for *D*. *sargus* and other fished species in the NT zone, but not in the PR and NR zones [36,37]. Taking into account that the movement behaviour of *D*. *sargus* does not change among zones, these results suggest that the restriction of fishing activities in the partial reserve has a limited benefit on fish populations and hence, the low efficiency of partially protected zones for the full recovery of fish populations [37,62,63].

Another consequence of the territoriality of the adult individuals of *D*. *sargus* is a very limited capacity to spillover. During the study period a very low number of cross-boundary movements were detected, mostly between the PR and NR zones, and they were linked to very specific ecological conditions (e.g. storms, spawning). Moreover, most of the individuals that were translocated failed to return from the NT zone to the PR. On continuous rocky habitats, *D*. *sargus* has shown large homing movements, and individuals traveling up to 600 and 900 m to their original capture zone have been reported [29,32]. Here, we hypothesize that the sand gap between the Medes Islands and the Montgrí massif rocky bottoms may be acting as an impediment to the movements of *D*. *sargus*. Indeed, habitat discontinuities have been noted to act as partial barriers for several coral reef species [8,64]. However, it is interesting to note that the sandy gap between the Medes islands and the nearby zone is not an obstacle for the movement of other species, such as the herbivorous fish *Sarpa salpa* [42].

### Extraordinary movements

Despite being strongly sedentary, *D*. *sargus* demonstrated the ability to undertake considerable movements to areas outside their ordinary depth-range and HR. Those movements, which were generally quick (lasting less than a day), were generated by specific physical (waves) and biological (spawning) factors. Extreme climatic events, such as severe storms, can act as mobilizing agents for benthic fish. During those extreme events *D*. *sargus* left its preferred shallow habitat, which was highly exposed to the wave action, and moved to areas where the hydrological conditions were less intense. In a parallel study, Pagès *et al*. [65] described movements of *S*. *salpa* to deep sheltered areas during the extreme storm of 2008. The same escaping behaviour has been also described for several tropical fish species [66,67]. These movements allow fish populations to endure the disturbances without suffering significant population losses [65]. Moreover, severe storms can relocate the HR of some of the individuals into new zones [66], and may be a mechanism by which sedentary species could generate adult spillover from an MPA to adjacent zones.

Our study also provides fundamental knowledge on the spawning behaviour of *D*. *sargus*. The spawning of this species is triggered by a change in the thermal regime, specifically related to the increase of the seawater temperature immediately after the winter minimum [53,54]. We observed that during this period, spawning movements happened in several pulses over a few days, in which *D*. *sargus* visited deep spawning areas. Most of those areas were located within the computed HR, but some individuals also travelled further distances up to 600 m away from the edge of their HR (1 km away from the centre of their core area). However, those movements seemed to have a limit, as none of the individual from the NT zone left the fully protected area, and none of the individuals from the PR and NR crossed to the NT zone. Similar spawning movements of the same magnitude (1–2 km) were reported for this species by Di Lorenzo *et al*. [32].

As the observed spawning movements occurred synchronously and during short periods of time, we hypothesize that *D*. *sargus* forms FSAs. Two types of FSAs have been described depending on their frequency, length of time, site specificity, and the distance travelled by individuals [10]. In ‘resident’ FSAs, individuals move to the spawning sites from relatively small and local areas in short migrations of few hours or less. They usually happen at a specific time over several days, and last for only few hours. ‘Transient’ FSAs, in contrast, are characterized by longer migrations from relatively larger areas, to specific spawning sites where the aggregation persists for longer periods of several days or weeks. The spawning movements that we have observed in *D*. *sargus* involved individuals moving small distances from their core HR areas to spawning sites. Therefore, we suggest *D*. *sargus* aggregations be considered as resident FSAs.

Aggregating behaviour of *D*. *sargus* during spawning has gone unnoticed until now, but it can be an important issue for the conservation of the species. To better understand the population dynamics, it is important to determine if the captures of *D*. *sargus*, by both artisanal and industrial fisheries, increase during the breeding season. Moreover, this fundamental gap of knowledge in such an abundant and widespread species raises new questions about the spawning behaviour of other, less common, species and highlights the need to further study the behavioural ecology of small Mediterranean fishes. Repetitive fishing activities on predictable FSAs can cause severe damage and the collapse of fish populations [13,14]. FSAs might be a common strategy among coastal fish species in the Mediterranean, therefore knowing whether the species generate FSAs, and their timing and location is essential when deciding the placement, size and shape, and protection-levels of MPAs.

## Supporting Information

S1 Fig Results of the signal range-tests performed in the study site. The dots represent the mean (±SE) probability of tag detection at increasing distances from acoustic receivers.
(PDF)

S2 Fig Spatial chronogram plots for each tagged *Diplodus sargus* individual. (PDF)

S3 Fig **Distribution of phase-transition values (PT) calculated for the hourly reception number between consecutive day-night (A) and day-day (B) phases, for each *Diplodus sargus* individual.** Lower and upper boundaries of the boxes represent the first and third quantiles of value distributions. Significant results of the Wilcoxon signed-rank tests against the null hypothesis of a median of zero are expressed by the symbols above the boxes; +: p < 0.05; *: p < 0.01.
(PDF)
