# Supplementary figures and images for: Ordinary and Extraordinary Movement Behaviour of Small Resident Fish within a Mediterranean Marine Protected Area
Source: PLoS One. 2016 Jul 20;11(7):e0159813. doi: 10.1371/journal.pone.0159813 (PMC4954665; doi:10.1371/journal.pone.0159813)

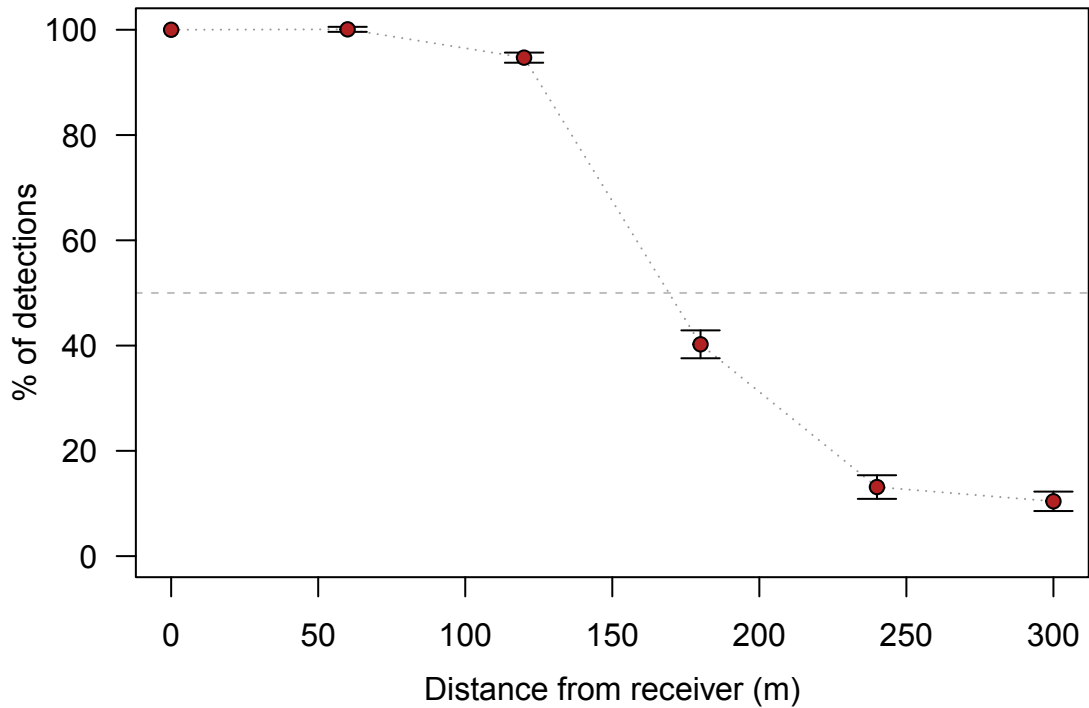

Supplement: S1 Fig — The dots represent the mean (±SE) probability of tag detection at increasing distances from acoustic receivers. (PDF) [file pone.0159813.s001.pdf]

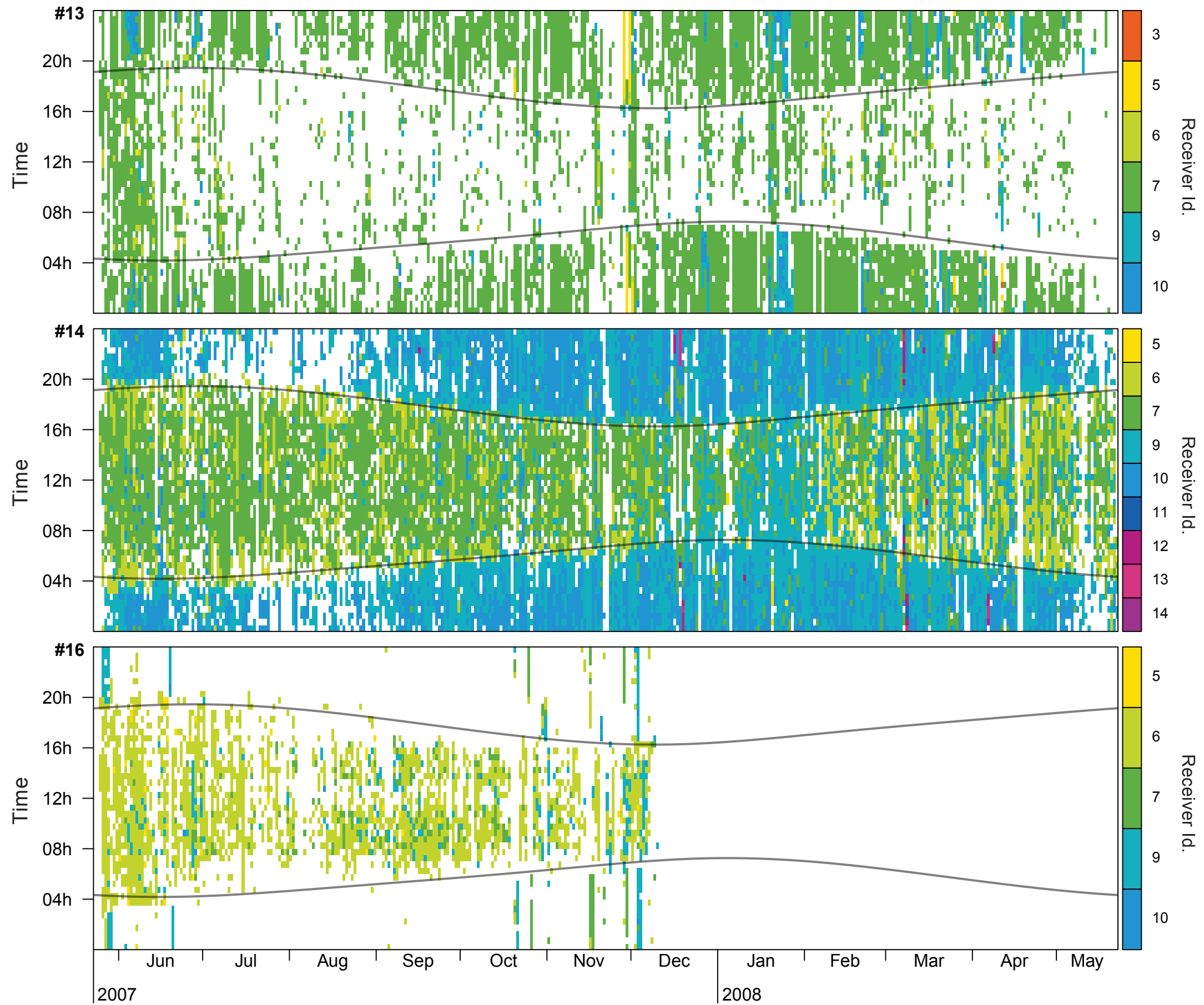

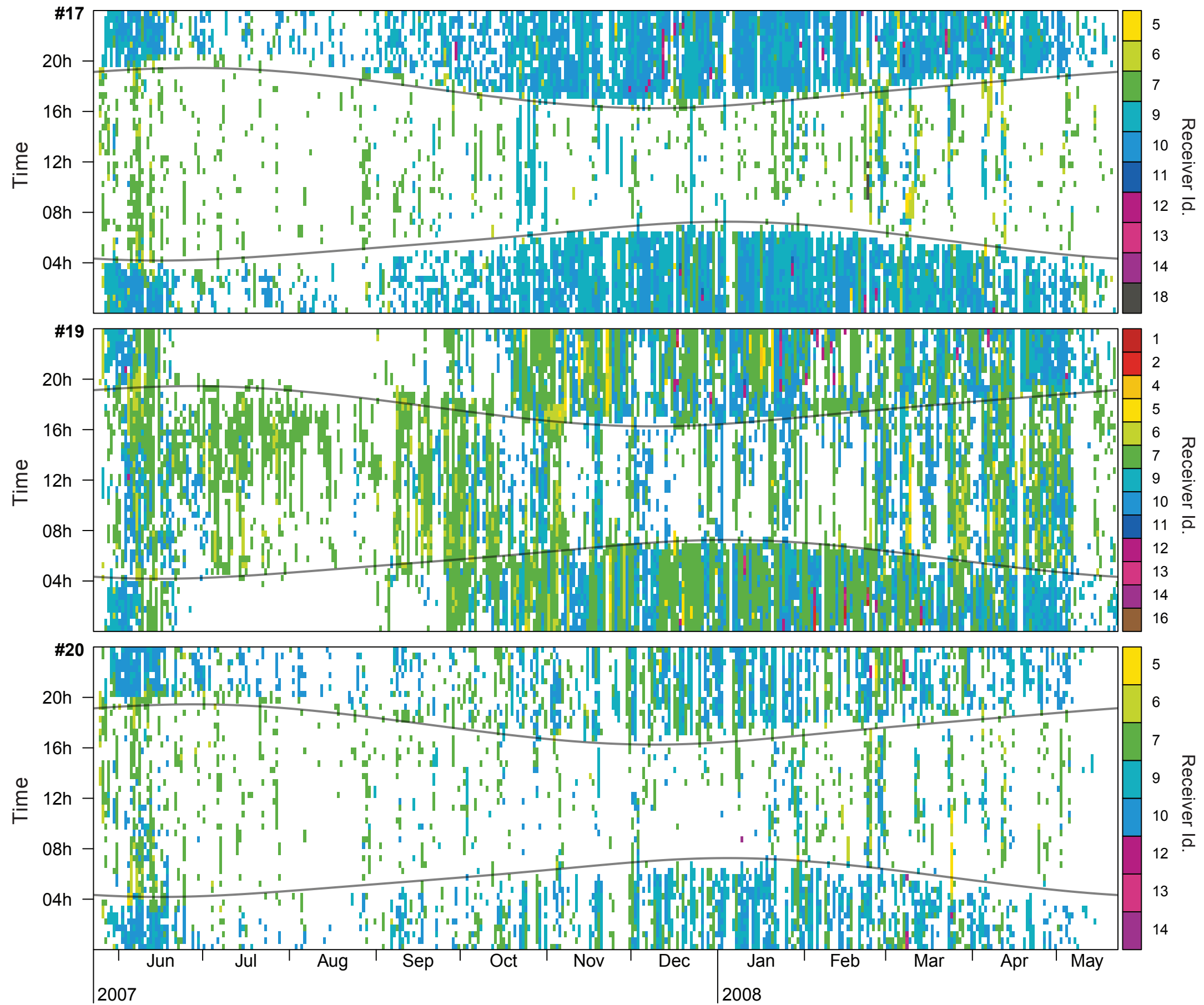

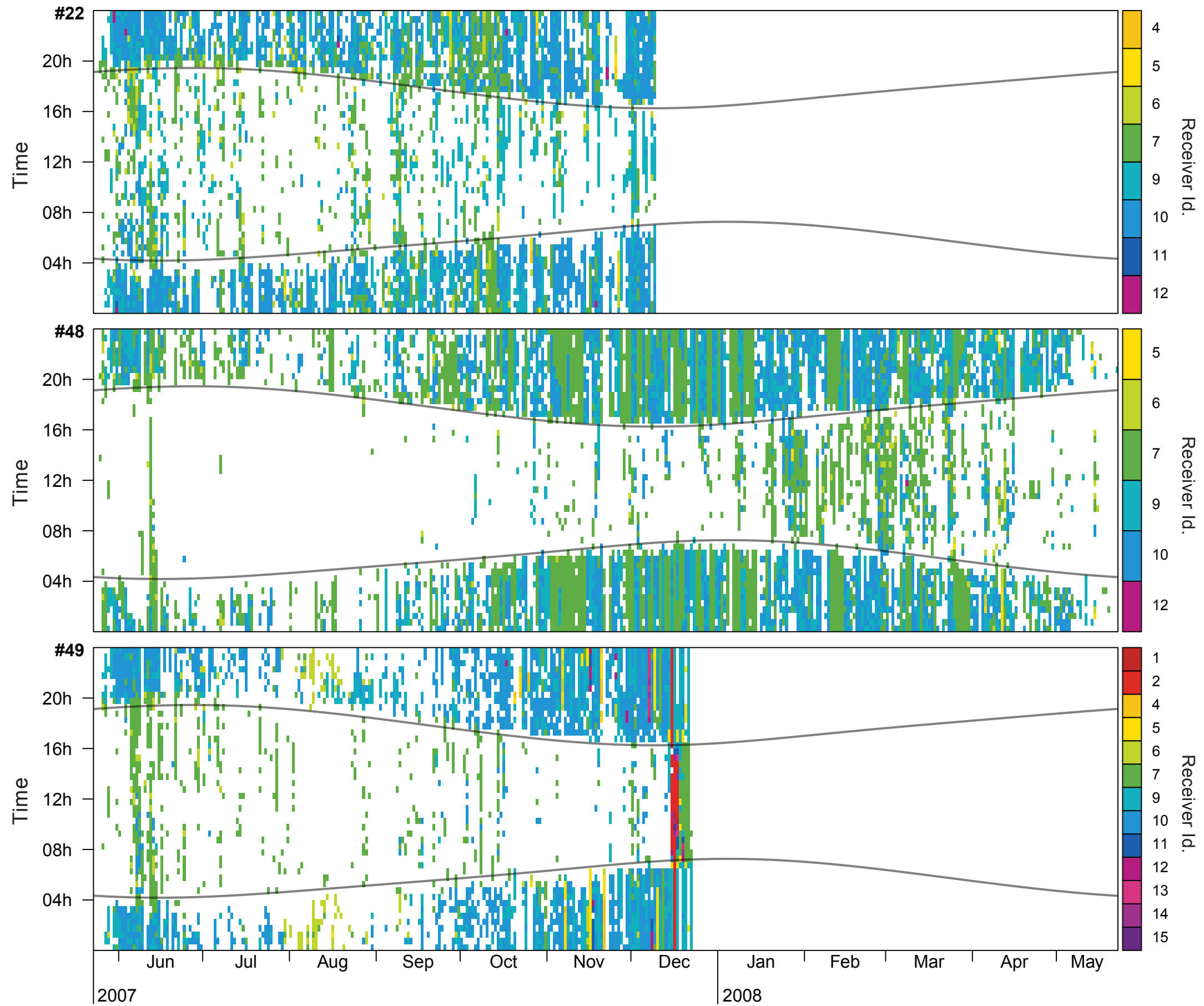

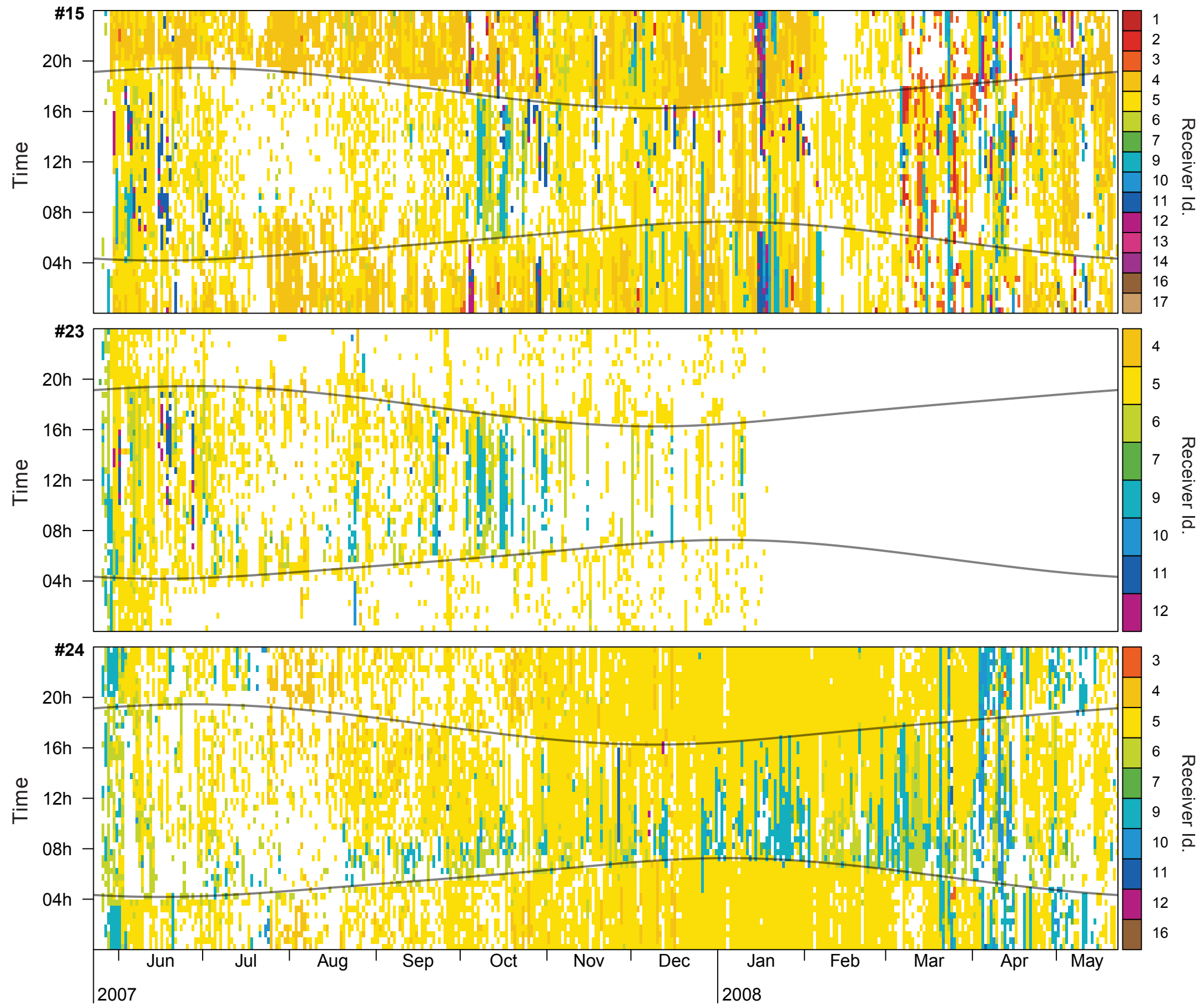

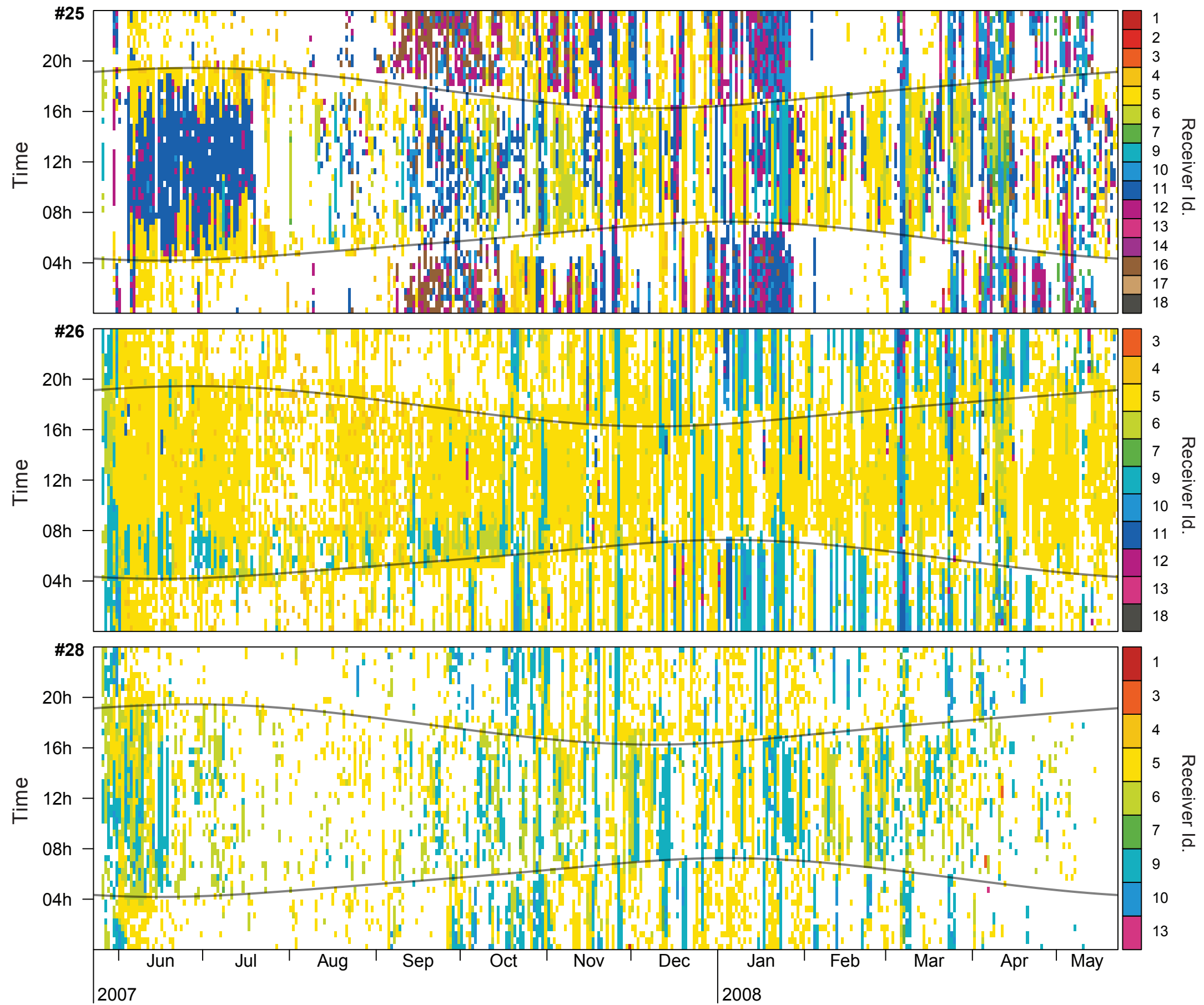

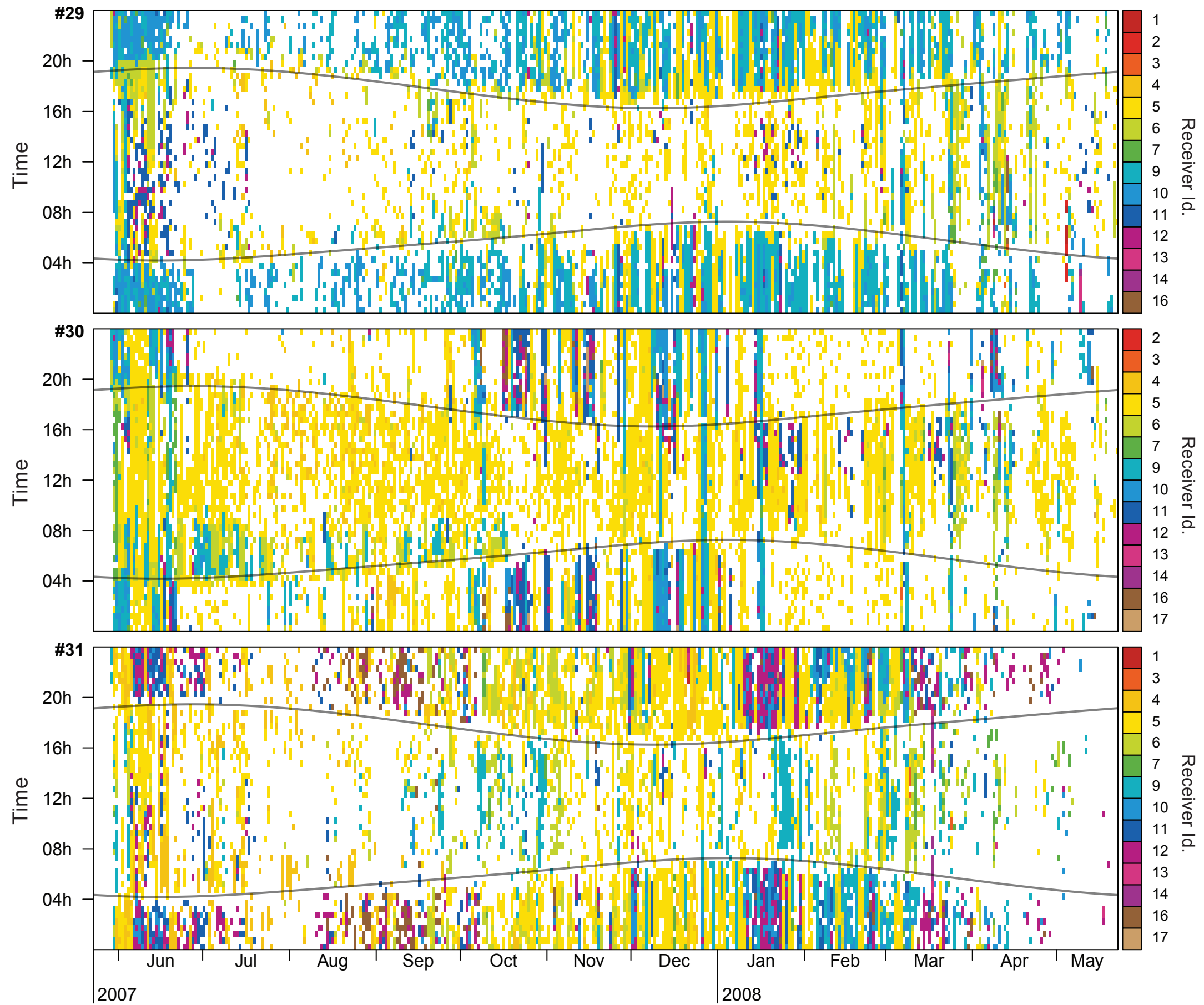

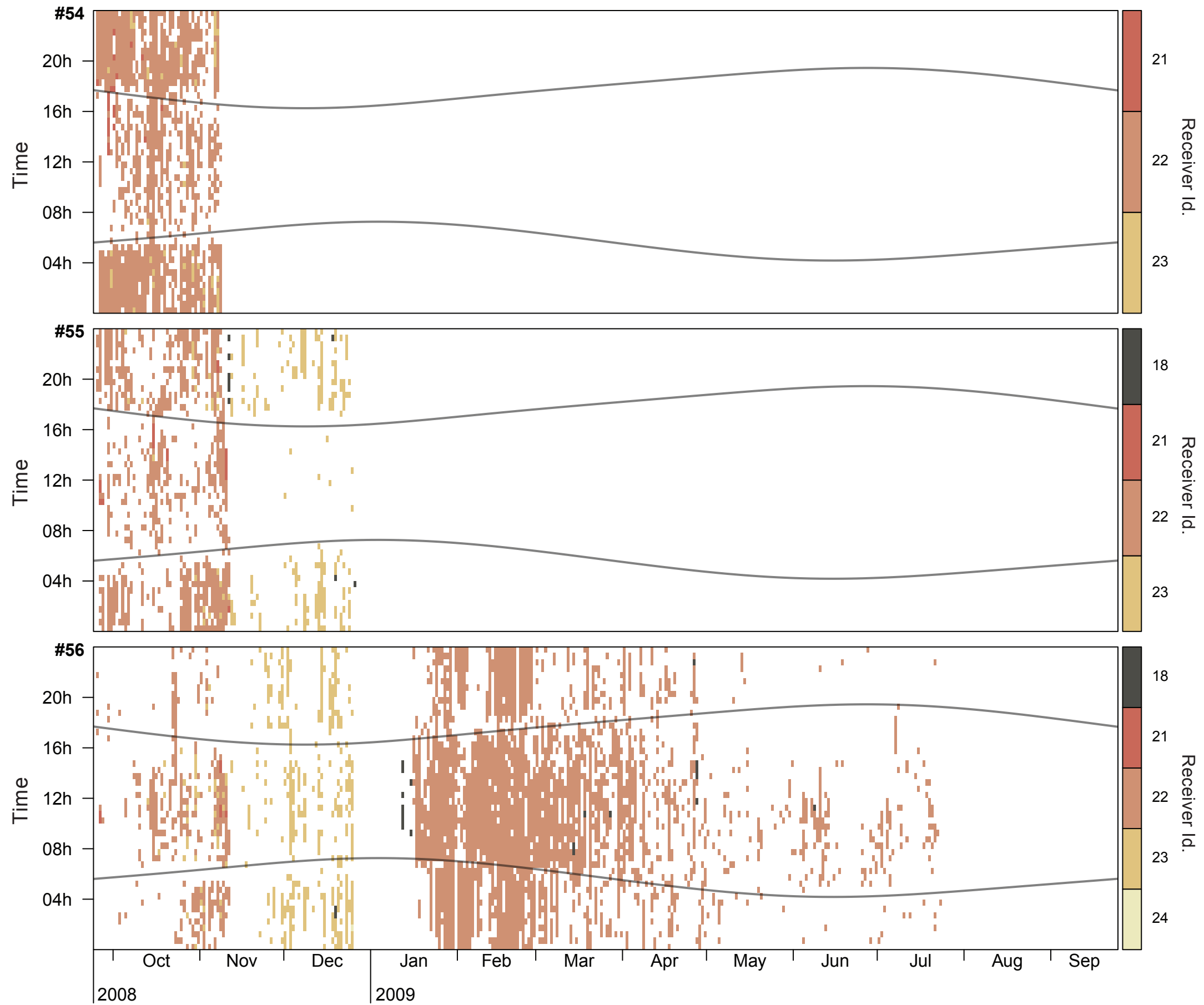

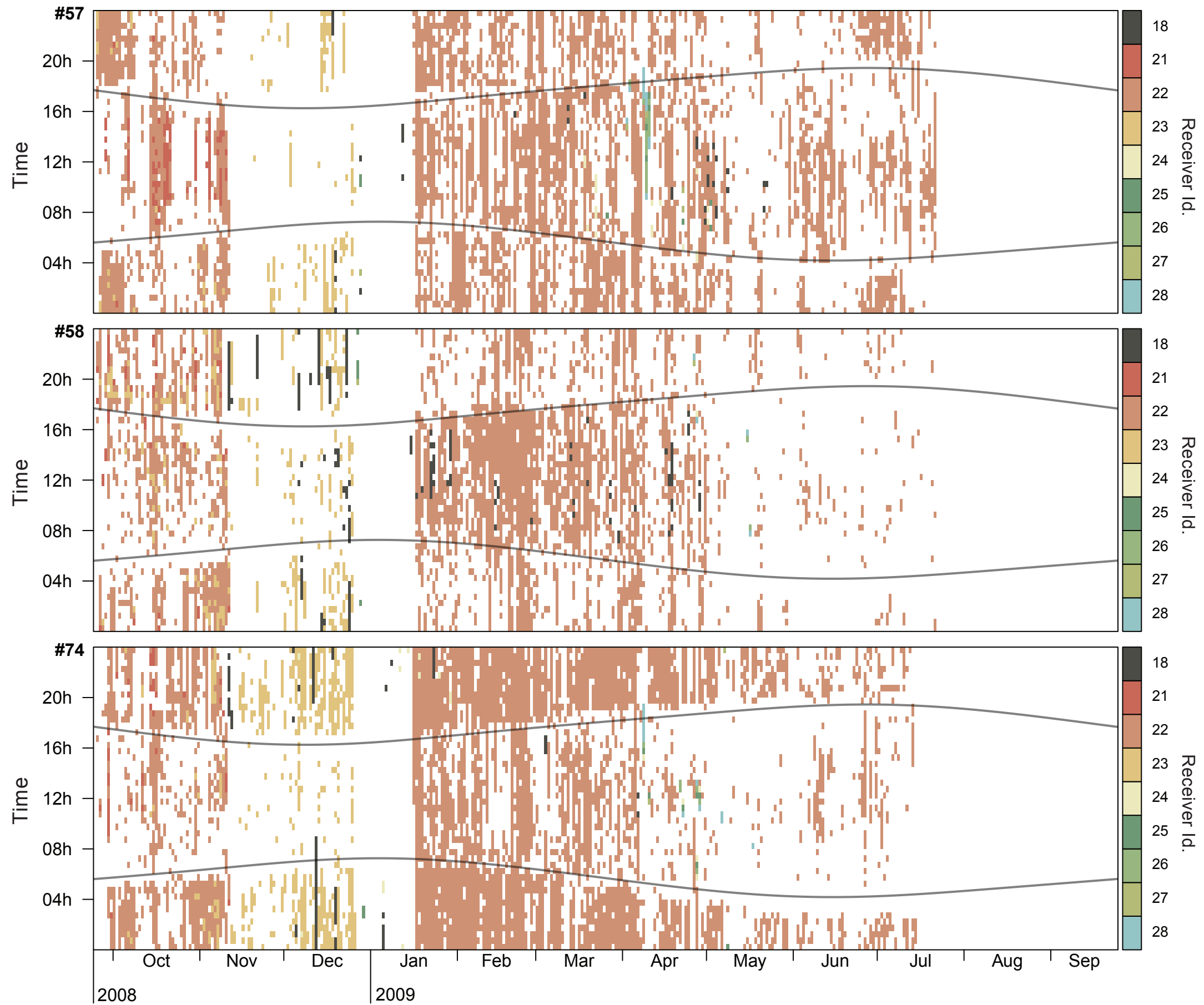

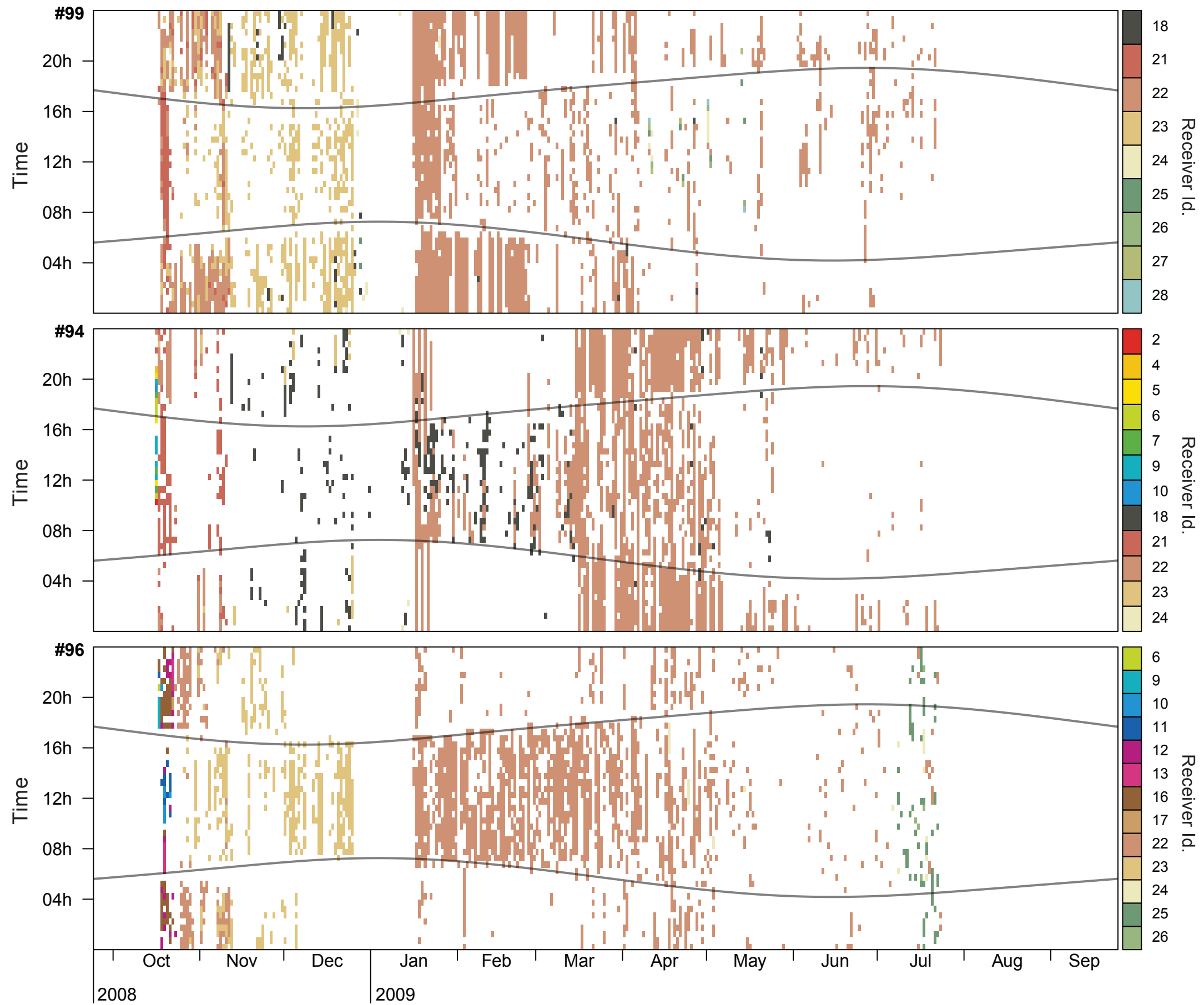

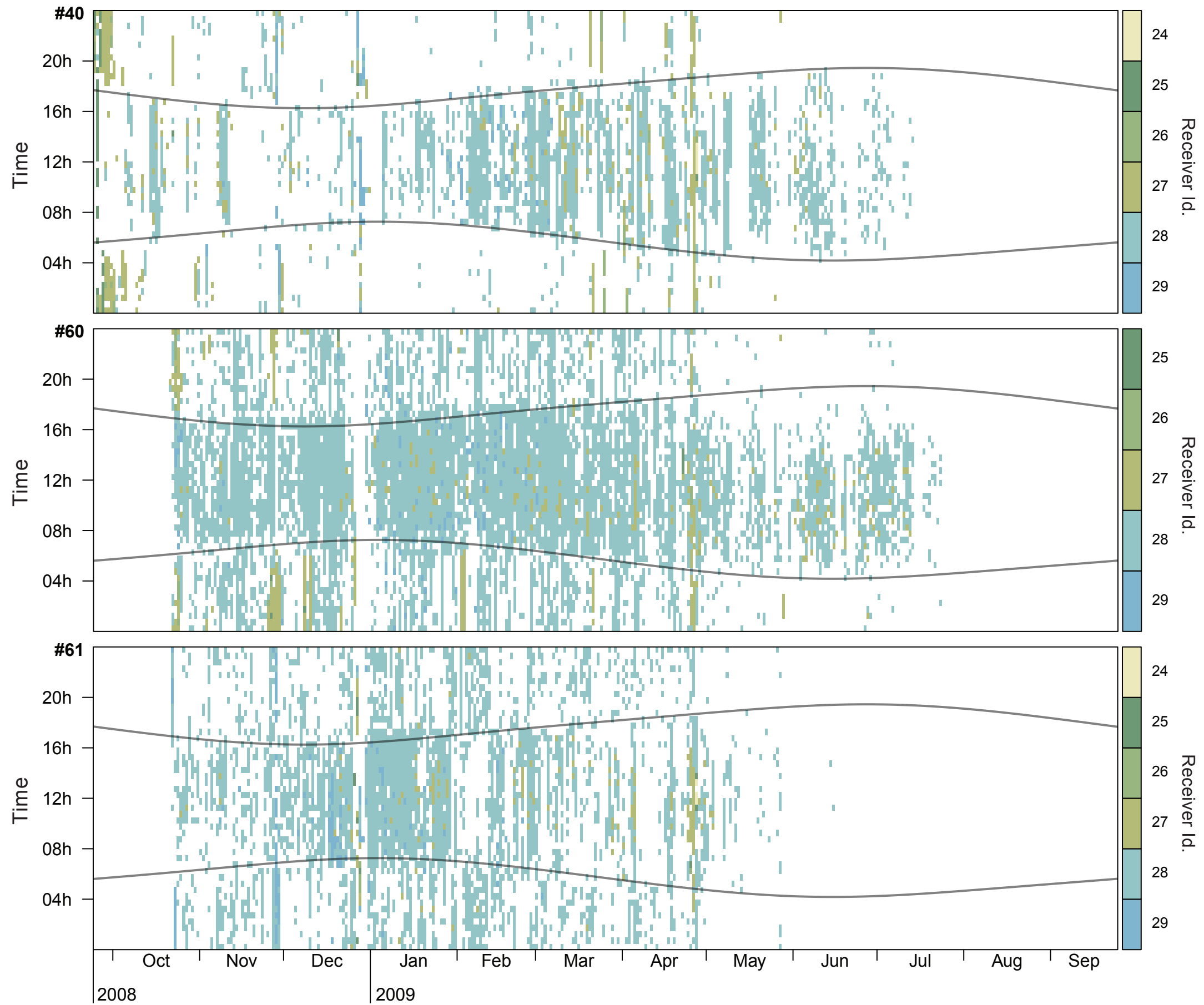

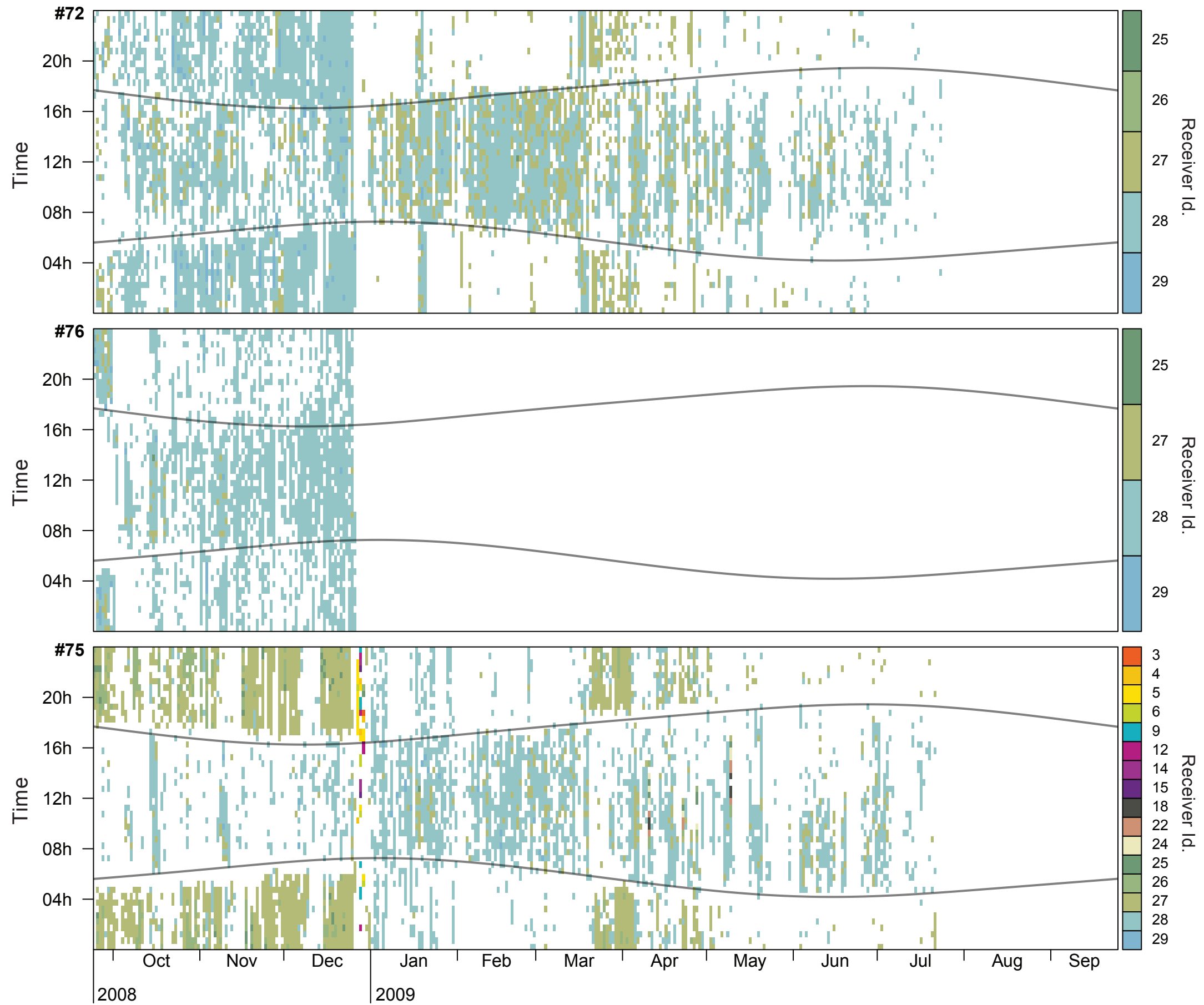

Supplement: S2 Fig — (PDF) [file pone.0159813.s002.pdf]

**A**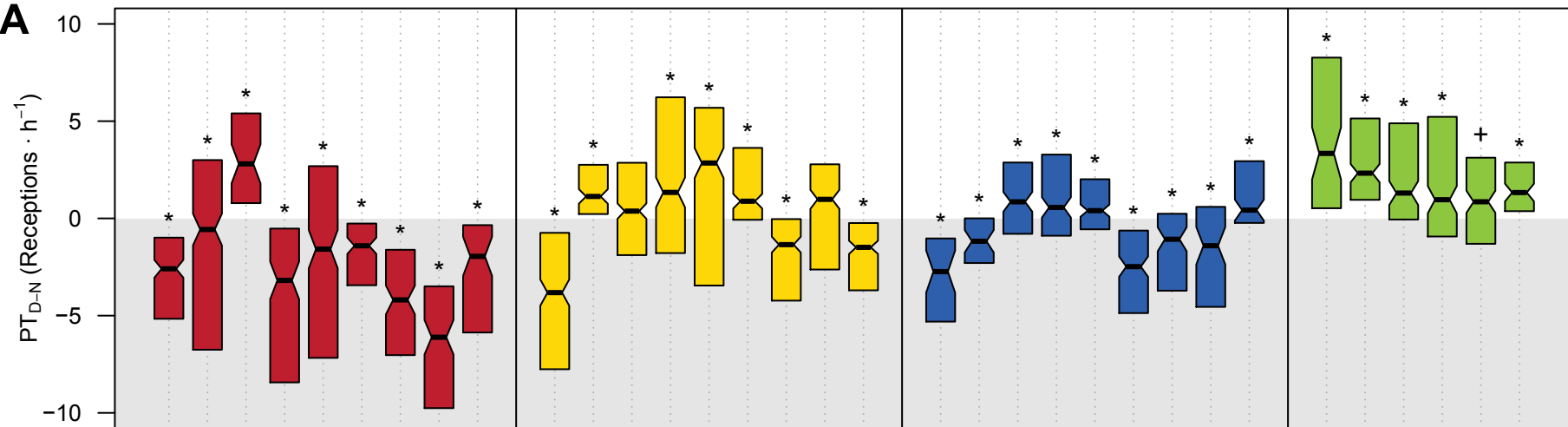**B**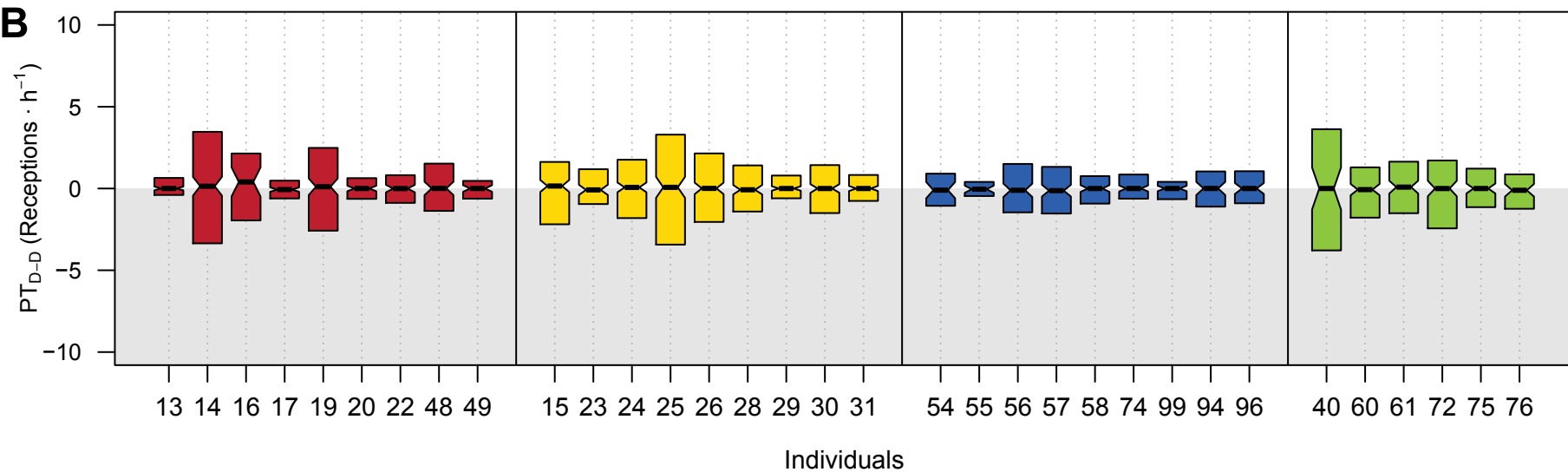

Supplement: S3 Fig — Distribution of phase-transition values (PT) calculated for the hourly reception number between consecutive day-night (A) and day-day (B) phases, for each Diplodus sargus individual. Lower and upper boundaries of the boxes represent the first and third quantiles of value distributions. Significant results of the Wilcoxon signed-rank tests against the null hypothesis of a median of zero are expressed by the symbols above the boxes; +: p < 0.05; *: p < 0.01. (PDF) [file pone.0159813.s003.pdf]
